# Comparative transcriptome analysis of *Rheum australe*, an endangered medicinal herb, growing in its natural habitat and those grown in controlled growth chambers

**DOI:** 10.1038/s41598-020-79020-8

**Published:** 2021-02-12

**Authors:** Deep Mala, Supriya Awasthi, Nitesh Kumar Sharma, Mohit Kumar Swarnkar, Ravi Shankar, Sanjay Kumar

**Affiliations:** 1 Biotechnology Division, Council of Scientific and Industrial Research-Institute of Himalayan Bioresource Technology, P.O. Box 6, Palampur, H.P 176061 India; 2 Academy of Scientific and Innovative Research (AcSIR), Ghaziabad, Uttar Pradesh 201002 India; 3 Studio of Computational Biology and Bioinformatics, Biotechnology Division, CSIR-Institute of Himalayan Bioresource Technology, Palampur, H.P 176061 India

**Keywords:** Molecular biology, Plant sciences

## Abstract

*Rheum australe* is an endangered medicinal herb of high altitude alpine region of Himalayas and is known to possess anti-cancerous properties. Unlike many herbs of the region, *R. australe* has broad leaves. The species thrives well under the environmental extremes in its niche habitat, therefore an understanding of transcriptome of *R. australe* to environmental cues was of significance. Since, temperature is one of the major environmental variables in the niche of *R. australe*, transcriptome was studied in the species growing in natural habitat and those grown in growth chambers maintained at 4 °C and 25 °C to understand genes associated with different temperatures. A total of 39,136 primarily assembled transcripts were obtained from 10,17,74,336 clean read, and 21,303 unigenes could match to public databases. An analysis of transcriptome by fragments per kilobase of transcript per million, followed by validation through qRT-PCR showed 22.4% up- and 22.5% down-regulated common differentially expressed genes in the species growing under natural habitat and at 4 °C as compared to those at 25 °C. These genes largely belonged to signaling pathway, transporters, secondary metabolites, phytohormones, and those associated with cellular protection, suggesting their importance in imparting adaptive advantage to *R. australe* in its niche.

## Introduction

High altitude (HA) environment is characterized by low partial pressure of gases, low-temperature, large fluctuations in diurnal temperature, high wind velocity, limited water and nutrient supply, solar radiations with higher fraction of ultraviolet (UV) rays, and narrow time window for growth and development. These are some of the factors which govern species diversity and distribution according to altitude^1,2^. There are many species adapted to HA alpine region by reprogramming their gene expression, enzyme activities, and metabolite concentrations^3^. *Rheum australe* D. Don (6–12 bivalent with 2n = 44^4,5^, synonym: *Rheum emodi* Wall. ex Meissn.) is one such species of family “Polygonaceae” that grows luxuriously in the grassy or rocky slopes of the Himalayas at an altitude of 3000–4200 m above mean sea level (amsl) across India, Nepal, and Bhutan^6,7^. In India, the species has been recorded from Jammu and Kashmir, Himachal Pradesh, Uttarakhand, Sikkim, and Arunachal Pradesh^6^. *R. australe* is a leafy perennial herb and is commonly known as “Himalayan Rhubarb” or red-veined pie plant^7^. Interestingly, in contrast to the small leaf features of many alpine herbs, *R. australe* exhibits an exceptionally unique broad leaf phenotype^8^.

*R. australe* has been a source of anthraquinones (emodin, chrysophanol, physcion, aloe-emodin, and rhein), stilbenoids (piceatannol, resveratrol) and their glycosides, and flavonoids which have various pharmacological properties^9^. Standardized extract of *R. australe* exhibits anti-cancerous properties which restricts the growth of human breast carcinoma (MDA-MB-435S), and liver carcinoma (Hep3B) cell lines^10^. Besides, the species yields a natural dye in cosmetics, textiles, and food industry^11^. Due to overexploitation of the species for various industrial purposes, overgrazing, habitat destruction due to human activities and shift of tree line due to climate change, the population of *R. australe* has shown severe depletion which brought it amongst the category of endangered plant species^7^.

During November to March, the habitat of *R. australe* in Indian Himalayas remains covered with snow. While during June to September (growing season) it experiences large variations in temperature which could be as high as 18.7 ± 3 °C and as low as 0.34 ± 3 °C. The annual temperature shows extremities with a maximum temperature touching 18.7 ± 3 °C and minimum going down to −18.2 ± 3 °C (data was recorded using data logger placed at Rohtang Pass, Kullu, Himachal Pradesh; unpublished data). Although *R. australe* has been investigated to study its chemical constituents, there is no data on the transcriptome of this species till date. Since temperature is one of the most dominant perceivable parameters at HA, the objective of the present work was to (i) compare the transcriptome profile of plant samples collected *in-situ* from its natural habitat with those grown at 4 °C and 25 °C in plant growth chambers and (ii) identify the common genes which show a similar trend of expression in the niche location and those exposed to 4 °C as compared to those at 25 °C. The present work identified a wide range of genes through RNA-Seq data generated on Illumina platform (Genome Analyzer IIx) which showed overexpression at the niche location and at 4 °C when compared to those at 25 °C and 28 genes were validated by quantitative real-time PCR (qRT-PCR). Analysis showed positive correlation between qRT-PCR and fragments per kilobase of transcript per million (FPKM) data.

## Results

### Reads generation and de novo assembly

**Figure 1:**
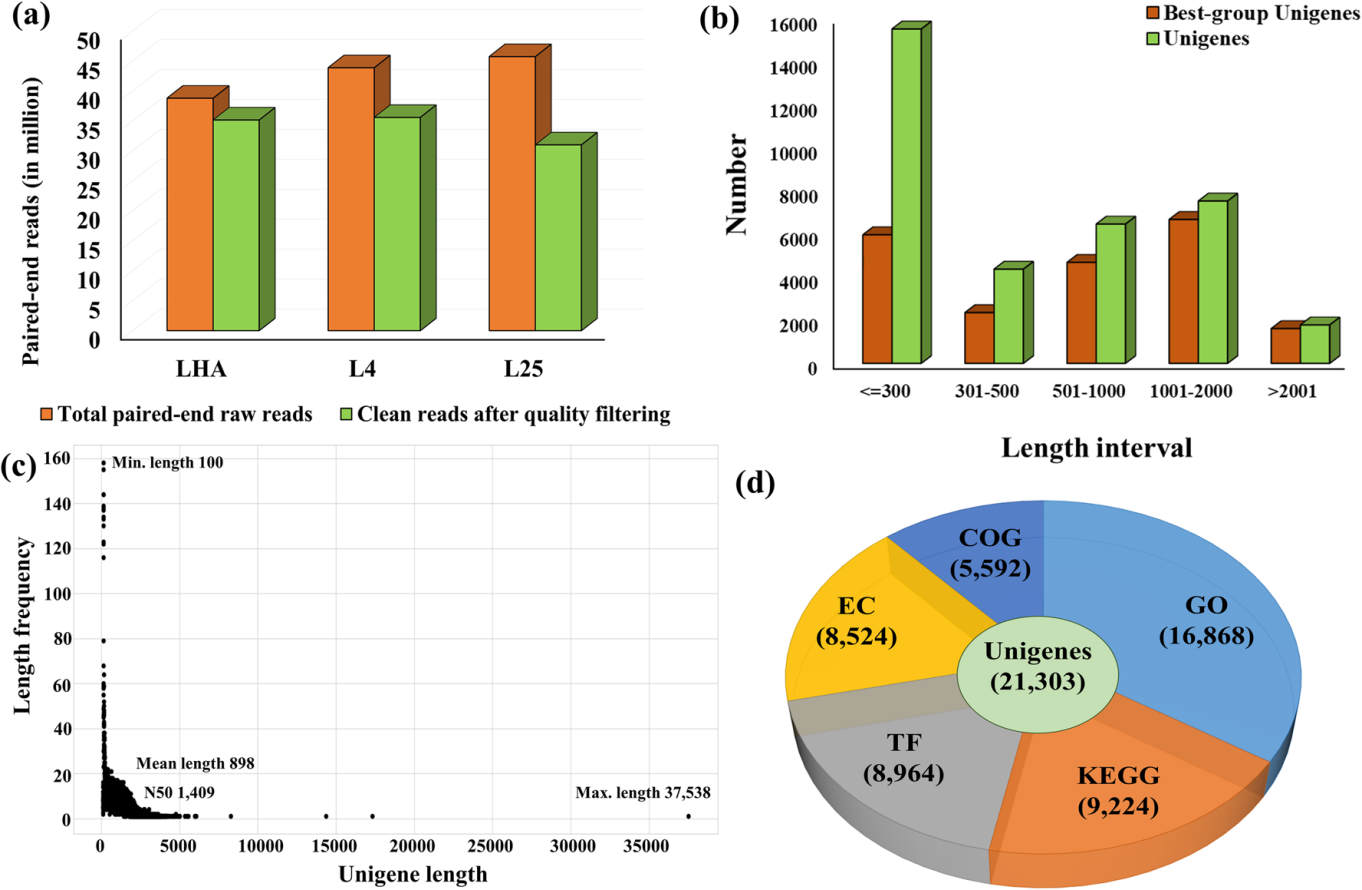
Overview of *Rheum australe* transcriptome read generation, assembly, and annotation of unigenes. **(a)** Paired-end (PE) read generation on Illumina Genome Analyzer IIx. X-axis indicates the analysis conditions where leaf samples were collected from the plants grown in growth chambers maintained at 4 °C (L4), 25 °C (L25), and the plants growing naturally in the niche location (LHA), y-axis indicates the number of PE raw and clean read. **(b)** Length distribution of unigenes and best group unigenes obtained from de novo assembly. **(c)** Best group length frequency distribution of unigenes. **(d)** The number of unigenes annotated against Non-Redundant (NR), Clusters of Orthologous Groups (COG), Gene Ontology (GO), Kyoto Encyclopedia of Genes and Genomes (KEGG) databases, and Enzyme Commission (EC).

Three cDNA libraries were generated from the plant growing naturally in the niche location and the plants grown in plant growth chambers maintained at 4 °C and 25 °C (Supplementary Fig. S1a-f). Leaf tissues collected from natural habitat at HA, 4 °C, and 25 °C were termed as LHA (leaf sample from plants grown in natural habitat at HA), L4 (leaf sample from plants grown at 4 °C), and L25 (leaf sample from plants grown at 25 °C), respectively. A summary of analysis conditions, de novo assembly, and annotation details are given in Supplementary Figure S2. A total of 3,88,05,544; 4,38,89,772; and 4,57,46,900 paired-end (PE) reads were generated from leaf sample of LHA, L4, and L25, respectively. Further, reads were reduced to 3,51,65,008; 3,55,94,352; and 3,10,14,976 after filtering for quality and contamination for LHA, L4, and L25, respectively (Fig. 1a). For de novo assembly, SOAPdenovo-Trans was run from k-mer size of 19 to 69 with the read length of 72 base pair (bp). A minimum cut off length of 100 bp and k-mer of 43 was found most appropriate for assembly. Gapfiller was used to fill the gapped regions to obtain the longer transcripts^12^. A brief summary of various steps involved in de novo assembly of *R. australe* transcriptome is given in Table 1.

**Table 1:** Overview of de novo assembly of *Rheum australe*.

Tool	k-mer	Average length	Maximum length (bp)	Total sequences	% Sequences greater than 1000 bp	Coverage	N50	GC%	Mapping %
SOAPdenovo-Trans	43	661.96	37,533	39,136	25.23	40.71	1277	45.40	56.55
Gap filler	43	661	37,538	39,136	25.25	43.75	1278	45.40	62.31
TGICL-CD-Hit clustering	43	683	37,538	35,679	26.18	43.81	1271	45.39	59.94
Best-group clustering	43	898	37,538	21,303	38.97	60.05	1409	42.59	55.18

### Sequence clustering, homology search, and assembly validation

A total of 39,136 primarily assembled transcripts were generated from the pooled data in which 25.23% transcripts had a length greater than 1,000 bp. All unigenes were longer than 100 bp. Hierarchical clustering with TGICL-CAP3 and CD-HIT tools with 90% similarity resulted in the reduction of a total number of assembled sequences from 39,136 to 35,679. Sequences were analyzed for homology search against the Non-Redundant (NR) protein database using BLASTX (E value-10^–5^). Significant blast hits were obtained for 23,569 sequences, while no hit was obtained for 12,110 sequences. Moreover, a total of 12,396 transcripts of known function and 11,173 transcripts of unknown function were identified from 23,569 sequences (Supplementary Table S1). There might be a possibility of multiple representatives of the same gene or isoforms which inflate the number of unique genes. Therefore, dissimilar sequence (DS) clustering was performed to identify such transcripts representing a single gene which caused reduction of total genes from 23,569 to 21,303 (Supplementary Table S1). The distribution of unigenes and best group unigenes is shown in Fig. 1b wherein, 26.18% of the unigenes (35,679) and 38.97% of the best group unigenes (21,303) were found longer than 1000 bp. The best group unigenes with a maximum length of 37,538 bp, an average length of 898 bp, and N50 length of 1409 bp were assembled (Fig. 1c, Table 1).

Sequences with no significant BLAST hit were further analyzed for possible functional domains by scanning against the conserved domain database (CDD). A total of 242 sequences were identified with significant conserved domains such as alpha-ketoglutarate decarboxylase (8.68%), transcription termination factor Rho (7.02%), and DNA polymerase III subunits gamma and tau (6.61%), etc. (Supplementary Table S1). Filtered reads of libraries were assigned to unigenes using the RNA-Seq by Expectation Maximization (RSEM) software and the expression levels of unigenes were calculated by FPKM. NR database queries of *R. australe* revealed low similarity with the sequences of *Chenopodium quinoa* (21.1%), *Beta vulgaris* subsp. *vulgaris* (19.3%), *Vitis vinifera* (14%), and *Spinacia oleracea* (13.1%) (Supplementary Fig. S3) thus, indicating its unique position in the evolution of angiosperms.

### Functional annotation and characterization of the unigenes

**Figure 2:**
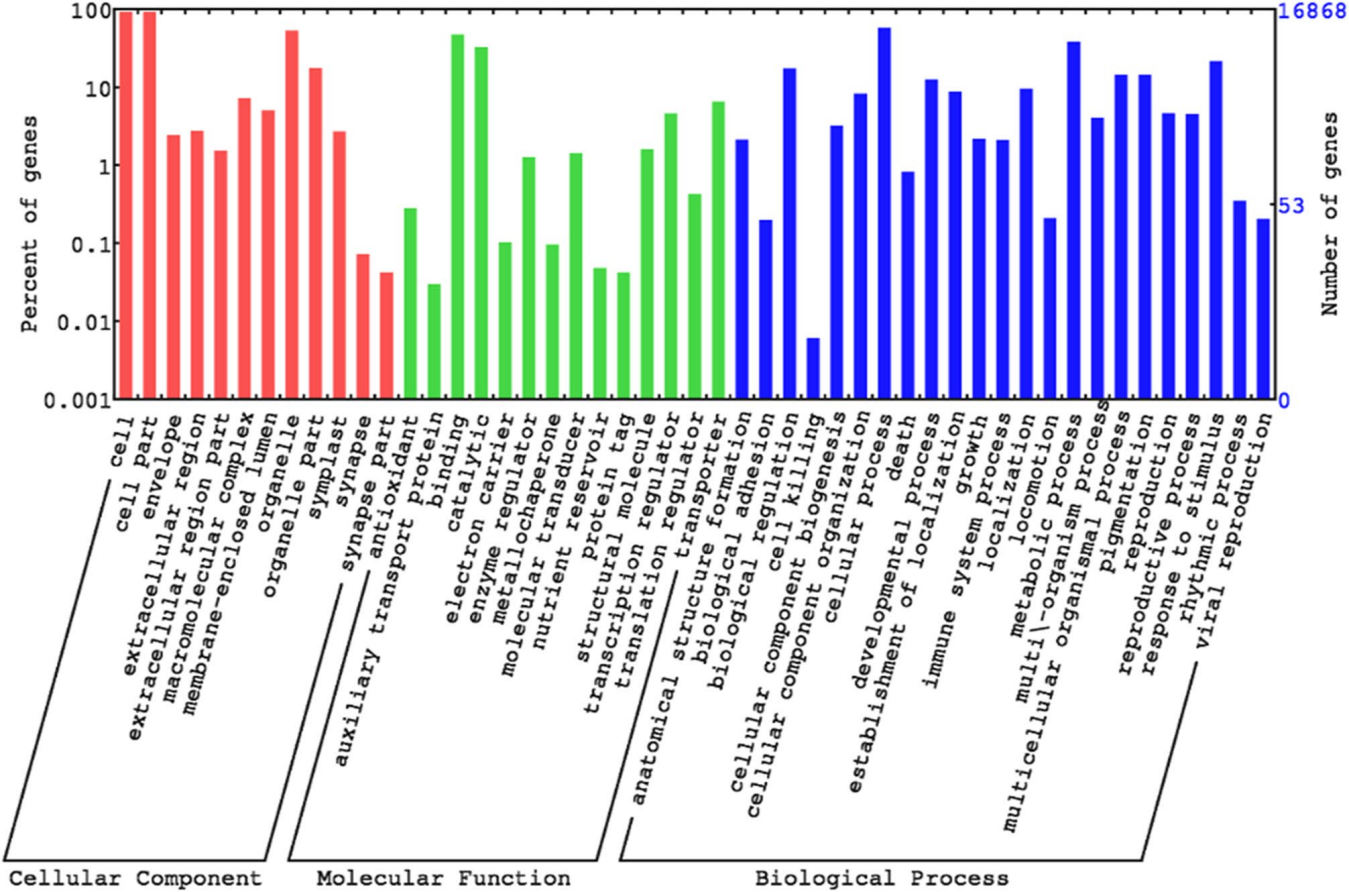
Functional classification of unigenes by Web Gene Ontology (WEGO). A total of 16,868 unigenes were annotated with the WEGO database for cellular component, molecular function, and biological process categories. X-axis indicates GO category and y-axis indicates the percent of transcripts.

Gene Ontology (GO) classification determines the potential function of unigenes. Therefore, 16,868 unigenes (79.18% of 21,303 unigenes) (Fig. 1d) were annotated under biological process (16,278; 76.41%), molecular function (15,147; 71.08%), and cellular component categories (16,070; 75.44%) (Supplementary Table S1). For a better understanding of gene functions at the macro level, Web Gene Ontology (WEGO) annotation plot was formed for GO functional classification (Fig. 2). In terms of biological processes, dominance of “metabolic process” and “cellular process” was identified. Among the molecular function category, “binding”, “catalytic”, and “transporter” activity were identified as the most significant group. In terms of cellular components, “cell”, “cell part”, and “organelle” were the most dominant components. Thus, GO classification indicated that the plants were under metabolically active growth state involving various enzymatic and transportation activities.

**Figure 3:**
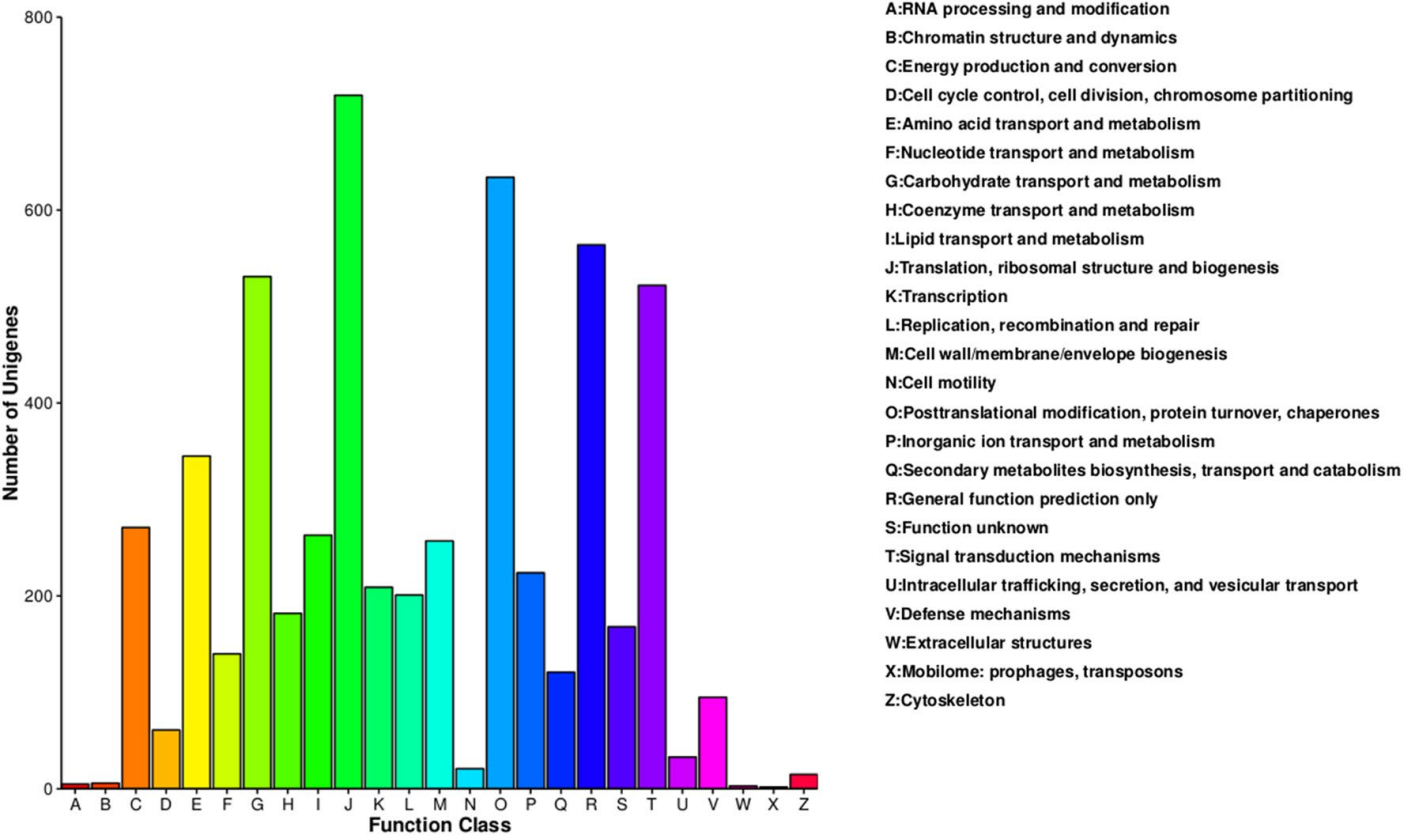
Classification of top 25 unigenes under Clusters of Orthologous Groups (COG). X-axis indicates COG function class and y-axis indicates the number of unigenes.

To identify the most abundant biological pathways and complex biological functional interactions in *R. australe*, 9224 unigenes (43.29% of 21,303) were annotated using the Kyoto Encyclopedia of Genes and Genomes (KEGG) database (Fig. 1d, Supplementary Table S1). Among them, the highest number of unigenes belonged to “plant hormone signal transduction” (18%) followed by “plant-pathogen interaction” (13%), and “ribosome” (10%) (Supplementary Fig. S4a). A total of 8,964 unigenes were annotated using the plant transcription factor (TF) database representing 42.07% of the transcriptome classified into 62 TF families (Fig. 1d, Supplementary Table S1). Among all the TF families in *R. australe*, “bHLH” (16%) was the most abundant category followed by “MYB_related” (10%), and “NAC” (9%) (Supplementary Fig. S4b). In terms of Enzyme Commission (EC) classification, 8,524 (40.01% of 21,303 unigenes) unigenes were classified (Fig. 1d, Supplementary Table S1). The most abundant unigenes were found in “non-specific serine/threonine protein kinase” (51%) enzyme class followed by “RNA helicase” (7%) (Supplementary Fig. S4c). An analysis of Guanine Cytosine (GC) content revealed the presence of approximately 62% of transcripts in the range of 41–50% of GC content (Supplementary Fig. S5). Total GC content of *R. australe* was 42.6% (Table 1) which is almost similar to that of *Arabidopsis thaliana* (dicot, 42.5%), a little lower than another Polygonaceae family plant, *Fagopyrum esculentum* ssp. *ancestrale* (45.9%)^13,14^, and much lower than rice transcriptome (monocot, 55%). The GC content of genes of an organism provides insights into evolution, gene structure, DNA thermostability gene regulation, and to identify evolutionary relationships among various species^14,15^. Assembled unigenes were evaluated at the protein level by “Clusters of Orthologous Groups” (COG) database and divided into 25 clusters. Among them, the cluster “J” for “translation, ribosomal structure, and biogenesis” (12.85% of 5,592 unigenes) represented the largest group, followed by cluster “O” for “post-translational modification, protein turnover, chaperone (11.33% of 5,592 unigenes), and cluster “R” for “general function prediction only” (10.08% of 5,592 unigenes) (Fig. 3). Results showed that the majority of unigenes were associated with protein synthesis, chaperon, and signaling.

### Analysis of differentially expressed genes (DEGs)

**Figure 4:**
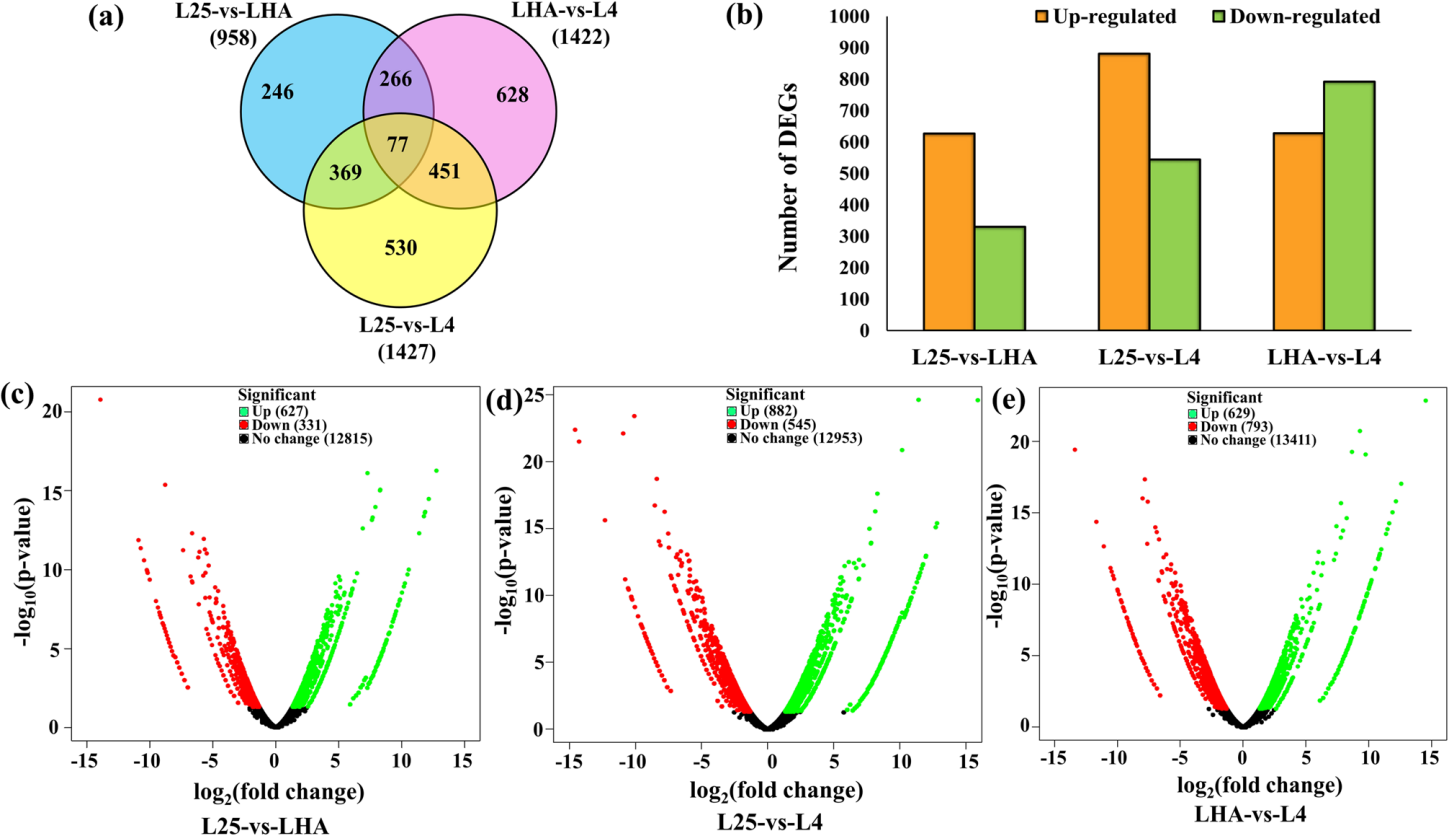
Venn diagram, histogram, and volcano plots of differentially expressed genes (DEGs). (**a)** Venn diagram shows DEGs expressed in each of the three L25-vs-LHA, L25-vs-L4, and LHA-vs-L4 conditions. Transcripts common in two or more comparative conditions are enclosed in the overlapping portion of the circles. **(b)** The number of DEGs in comparisons between pairs of libraries. X-axis indicates the comparisons between L25-vs-LHA, L25-vs-L4, and LHA-vs-L4; y-axis indicates the number of DEGs. **(c,d,e)** Volcano plots of the distribution of DEGs between L25-vs-LHA, L25-vs-L4, and LHA-vs-L4 respectively. X-axis shows the log_2_ fold change in gene expression. Y-axis shows the -log_10_ (p-value). The further from 0 on x-axis, the greater the change in expression and the higher on y-axis, the greater the significance. The green dots indicate up-regulated, red dots indicate down-regulated, and black dots indicate no change in expression in DEGs.

In order to find the DEGs among LHA, L4, and L25, a comparative analysis was performed as follows: (a) L25-vs-LHA_LHA_up_ (up-regulated DEGs in LHA as compared to L25), (b) L25-vs-LHA_LHA_down_ (down-regulated DEGs in LHA as compared to L25), (c) L25-vs-L4_L4_up_ (up-regulated DEGs in L4 as compared to L25), (d) L25-vs-L4_L4_down_ (down-regulated DEGs in L4 as compared to L25), (e) LHA-vs-L4_L4_up_ (up-regulated DEGs in L4 as compared to LHA), and (f) LHA-vs-L4_L4_down_ (down-regulated DEGs in L4 as compared to LHA). Significantly up- and down-regulated DEGs were identified using edgeR tool with log fold change (FC) ≥ 2 at a statistical significance level of p ≤ 0.05 and false discovery rate (FDR ≤ 0.05) (Supplementary Table S2). In the case of L25-vs-LHA, a total of 13,773 DEGs were identified of which 627 were up-, 331 were down-regulated, and 12,815 showed no change in expression in LHA as compared to that in L25 (Fig. 4a,b,c; Supplementary Table S2). In L25-vs-L4, out of 14,380; 882 were up-, 545 were down-regulated, and 12,953 showed no change in expression in L4 as compared to that in L25 (Fig. 4a,b,d; Supplementary Table S2). Under LHA-vs-L4 condition, 14,833 DEGs were obtained, wherein 629 were up- and 793 were down-regulated, while 13,411 showed no change in expression in L4 as compared to that in LHA (Fig. 4a,b,e; Supplementary Table S2). Further common up- and down-regulated DEGs were identified in LHA and L4 as compared to that in L25. Wherein, up-regulation of 276 (22.4%) common DEGs was found in LHA and L4 as compared to that in L25, while 161 (22.5%) common genes showed down-regulation under the above condition. Remaining 957 (77.6%) up- and 554 (77.5%) down-regulated genes did not exhibit any specific pattern (Supplementary Fig. S6). All DEGs were identified and annotated using BLASTX search tool and classified for functional categories and pathway assigned using GoSlim and KEGG. Comparative analysis between unigenes and DEGs showed that under biological processes, “response to stimulus” predominated (Supplementary Fig. S7a-d, Supplementary Table S3), while under molecular functions category, “transporter activity” was upregulated in L25-vs-LHA and L25-vs-L4, respectively (Supplementary Fig. S8a-d, Supplementary Table S3).

### Functional enrichment analysis of DEGs

Hypergeometric test was applied with Bonferroni correction at p ≤ 0.05 to identify the significant GO terms. In the GoSlim biological process, “secondary metabolic process” (p-value: 0.0406), “sterol biosynthetic process” (p-value: 0.0421), and “response to hormone stimulus” (p-value: 0.00346) were found highly significant in L25-vs-LHA. In L25-vs-L4, “response to stimulus” (p-value: 2.83e-07) and “aromatic compound biosynthetic process” (p-value: 0.0232) were found significant (Supplementary Fig. S9a-b, Supplementary Table S4). In the GO, “molecular function” category in L25-vs-LHA revealed a high level of up-regulated genes associated with “TF activity” (p-value: 0.00117), “cytokinin dehydrogenase activity” (p-value: 0.0155), and “caffeoyl-CoA O-methyltransferase” (p-value: 0.0378) activity. Later is associated with the enforcement of the plant cell wall, while in L25-vs-L4, “transporter activity” (p-value: 0.00199) was found highly significant (Supplementary Fig. S9c-d, Supplementary Table S4). KEGG pathway enrichment was performed using KEGG Automatic Annotation Server (KAAS) to gain insight into the significantly modulated biological pathways in DEGs. Top enriched up-regulated pathways in both L25-vs-LHA and L25-vs-L4 DEGs were “plant-pathogen interaction”, “plant hormone signal transduction”, “starch and sucrose metabolism”, and “circadian rhythm”, while in L25-vs-LHA “photosynthesis” and “photosynthesis antenna proteins” were down-regulated and “ribosome” and “steroid biosynthesis” related genes showed down-regulation in L25-vs-L4 (Supplementary Table S5).

### Assembly validation against the expressed sequence tags (ESTs)

Assembled transcripts of de novo assembly were validated against available ESTs of *R*. *australe.* A total of 608 ESTs of *R. australe* were reported in NCBI dbEST. However, significant hits were observed for 515 sequences (84.7%) while no hit was obtained for 93 ESTs (15.29%). A total of 62.13% ESTs (320/515) had a coverage greater than 90% whereas 86.40% ESTs (445/515) had coverage greater than 50%. GO analysis of total assembled transcripts was performed and a total of 103 sequences were identified for term “cold”. Out of 103 sequences, a total of 90 transcripts were found for “response to cold”, nine for “cold acclimation”, and four transcripts were found for “cellular response to cold”. Similarly, GO analysis of ESTs identified four transcripts related to term “response to cold”.

### Validation of assembly and FPKM based expression of DEGs by qRT-PCR

**Figure 5:**
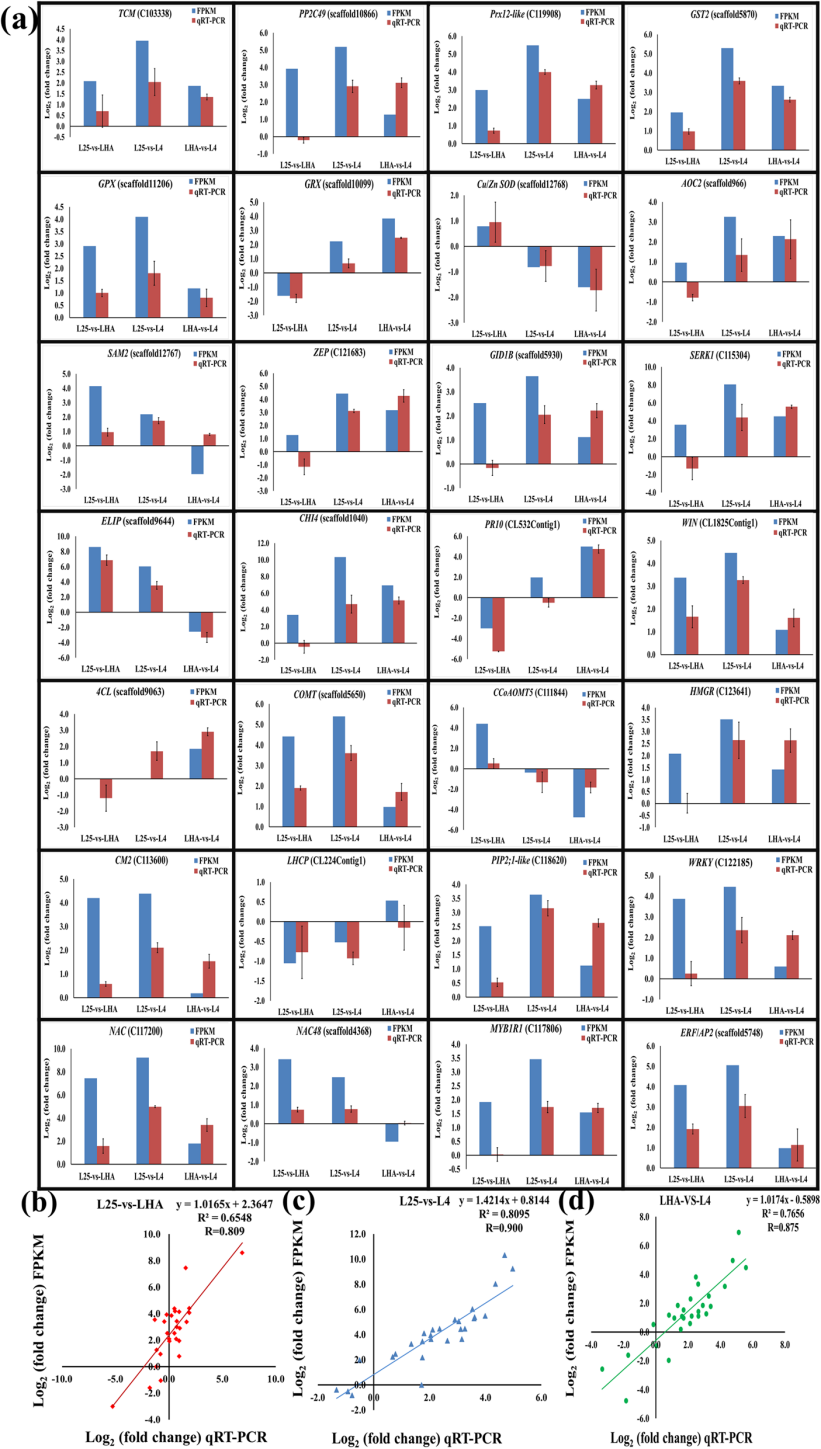
Validation of differentially expressed genes (DEGs) and correlation of gene expression. **(a)** Validation of FPKM based relative expression of 28 DEGs by qRT-PCR. X-axis indicates the conditions of the comparisons viz. L25-vs-LHA, L25-vs-L4, and LHA-vs-L4, whereas y-axis indicates the log_2_ fold change values of DEGs based on FPKM and qRT-PCR. Pearson correlation coefficient (R) was calculated between log_2_ fold change values of qRT-PCR (x-axis) and FPKM (y-axis) for **(b)** L25-vs-LHA, **(c)** L25-vs-L4, and **(d)** LHA-vs-L4.

In order to authenticate the reproducibility and confirm the validity of FPKM-based expression of RNA-Seq data, qRT-PCR was carried out for randomly selected 28 DEGs, which were identified in the top GO and KEGG pathways. The primers list for qRT-PCR analysis is given in Supplementary Table S6. The expression pattern of maximum genes was in accordance with each other and moderately correlated, however, some samples showed the obscure correlation in FPKM and qRT-PCR expression values (Fig. 5a). The Pearson correlation coefficient between log_2_ FC FPKM and qRT-PCR was 0.809 in L25-vs-LHA, 0.900 in L25-vs-L4, and 0.875 in LHA-vs-L4, indicating the reliability of the present RNA-Seq data (Fig. 5b,c,d).

### Identification of various common genes in LHA and L4

*R. australe* grows in extreme conditions of alpine region, which makes the species a valuable resource for bioprospecting genes. An analysis of DEGs in LHA and L4 as compared to L25 showed an abundance of genes associated with transporters (71), secondary metabolites (57), phytohormones (51), signaling (41), wounding (40), chaperones (39), antioxidants (30), DNA-repair system (14) (Supplementary Table S6) and TF (1,592) (Supplementary Table S7 and S8). *Ethylene response factor* (*ERF*), *MYB_related*, *NAC*, *bHLH*, and *WRKY* were major TF classes identified as DEGs in *R. australe* (Supplementary Fig. S10a–c).

## Discussion

**Figure 6:**
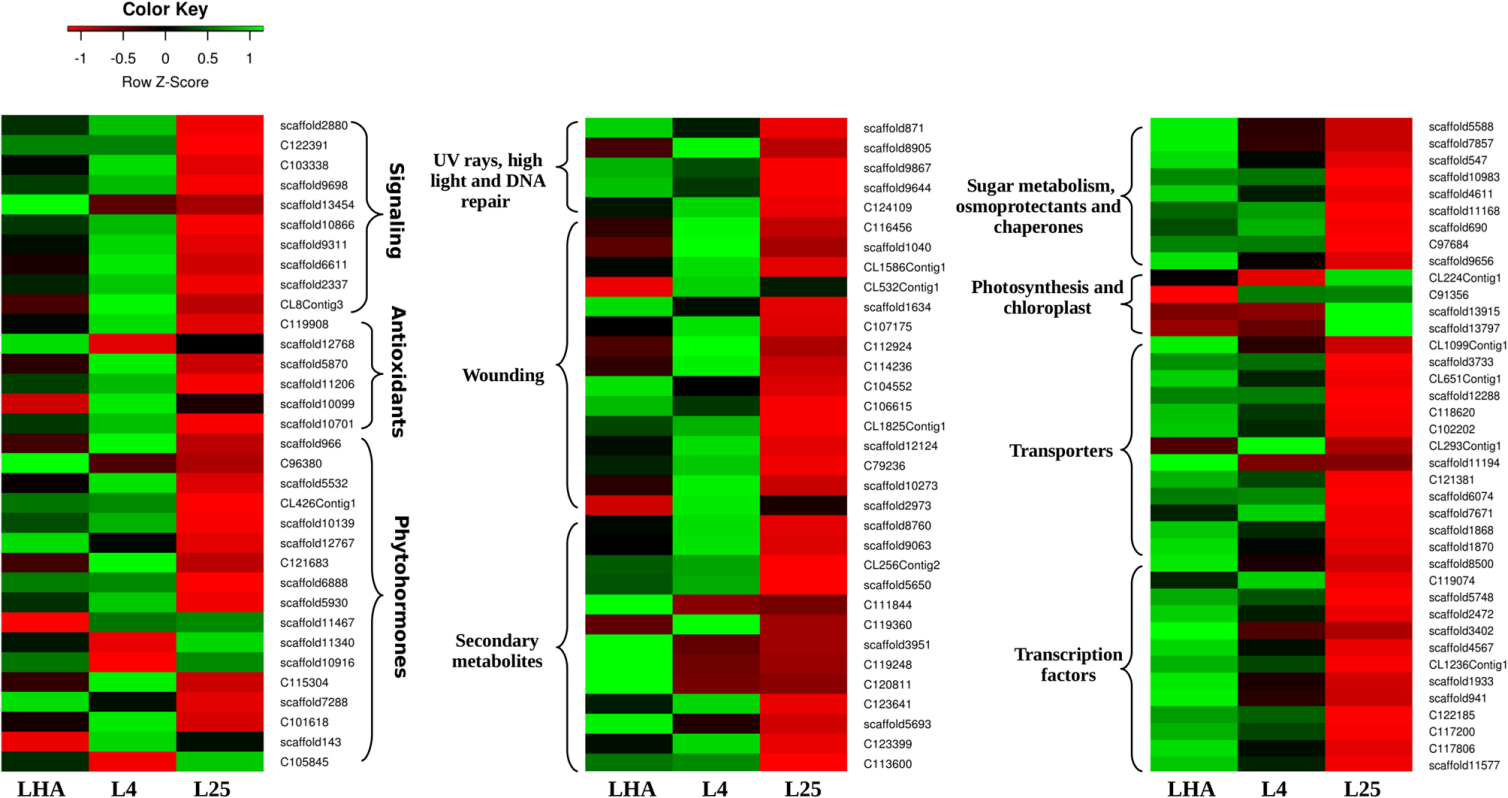
Heat map representation of differentially expressed genes (DEGs). The expression values are RNA-Seq FPKM values under three different conditions for 105 transcripts. Samples are represented in columns, whereas transcripts are shown in rows. The differences in expression level are shown in distinct colors. Green and red color indicates up- and down-regulation of DEGs, respectively. Gene description and annotation details are given in Supplementary Table S6. The heat maps were constructed using the gplot included in R package version vol. 2^117^.

One of the earliest responses of the plants under the fluctuating extreme environmental conditions is to influx Ca^2+^ from extracellular spaces into the cytosol. A correlation between cold-induced Ca^2+^ influx and plant acclimation has been shown in *Medicago sativa*^16^ and in *A. thaliana*^17^. In *R. australe*, *calcium-dependent protein kinase* (*CDPK*)*, calcineurin B-like protein-interacting protein kinase* (*CIPK*), *Ca*^2+^ *binding protein* (*TCM*), *calmodulin-binding protein, calmodulin-binding transcription activator* (*CAMTA*), and *mitogen-activated protein kinase* (*MAPK*) showed up-regulated expression in LHA and L4 as compared to that in L25 (Fig. 6). MAPKs can phosphorylate other kinases and/or various TFs. In *A. thaliana*, various abiotic stresses such as cold, heat, salt, UV, and osmotic-stress activated MAPK cascade^18^.

Beside MAPKs, protein phosphorylation through CDPKs and CIPKs enhanced cold tolerance in *Camellia japonica*^19^. Histidine kinase (HK), a transmembrane protein and wall-associated receptor kinases (WAKs) are involved in signal perception and transduction across the cellular membrane. In *R. australe*, *HK3, APRR1, ARR2*, *WAK1, WAK-like1*, and *WAK-like22* transcripts showed up-regulation in LHA and L4 (Fig. 6). HKs are involved in cytokinin (CK) and ethylene (ET) signaling, cold, osmotic perception, megagametophyte development, and resistance against bacterial and fungal infection in *A. thaliana*^20^. Similarly, WAKs are transmembrane proteins that bind directly to pectin. These are involved in cell expansion, plant development, and various other biotic and abiotic stresses in plants^21^. A large number of up-regulated *WAKs* were identified in response to cold stress^22^. Hence, enhanced expression of transcripts for Ca^2+^ signaling and transmembrane receptor proteins indicated a quick and diverse signaling mechanism in *R. australe* at 4 °C and in its natural habitat*.*

Unfavorable conditions produce reactive oxygen species (ROS), which also acts as signaling molecules and are involved in the plant stress acclimation. Various antioxidant enzymes regulate ROS concentration in the cell^23^. In the present work, *peroxidase* (*PRX*), *glutathione transferase*, *glutathione S-transferase* (*GST*)*, glutathione-peroxidase* (*GPX*), *glutaredoxin* (*GRX*), and *monodehydroascorbate reductase* (*MDAR*) showed up-regulated expression in both LHA and L4, while *superoxide dismutase* (*SOD*) was up-regulated in LHA and its down-regulation was observed in L4 (Fig. 6). PRX, a classIII enzyme (EC:1.11.1.7) is involved in the plant defense by phytoalexin and in the metabolism of ROS^24^. SOD was reported to reduce cold injury in cold acclimatized rice and spinach^25,26^. Transgenic plants overexpressing *GST* exhibited tolerance to salt and drought stress in *A. thaliana*^27^, whereas this overexpression enhanced germination rate and growth in response to low-temperature in rice^28^. Therefore, ROS mediated signaling could activate antioxidant enzymes and might be responsible for imparting stress tolerance to *R. australe* in the niche location as well as at 4 °C.

Phytohormones are involved in modulating the response of plants to different stresses. Three phytohormones, jasmonic acid (JA), salicylic acid (SA), and ET are known to regulate plant defense responses against various biotic and abiotic stresses^29^. Lipoxygenase (LOX) and jasmonate O-methyltransferase (JMT) are involved in JA biosynthesis and methylation of jasmonate into methyl jasmonates (MeJAs), respectively. MeJAs modulate various growth and developmental processes, defense mechanism, and impart chilling tolerance in plants^30,31^. In case of SA, salicylate carboxymethyltransferase (SAMT) converts SA to methyl ester (MSA) of SA by methylation. MSA, a volatile compound, triggers defense responses and acts as a chemoattractant for moth pollinated flowering plants^32^. In *R. australe*, *allene-oxide cyclase2* (*AOC*), *LOX*, *JMT*, and *SAMT* were up-regulated in LHA and L4 as compared to that in L25.

Synthesis of ET begins with the production of its precursor, S-adenosylmethionine (SAM) which is catalyzed by SAM synthase from methionine, while 1-aminocyclopropane-1-carboxylate oxidase (ACO) converts 1-aminocyclopropane-1-carboxylic acid (ACC) to ET and becomes a rate-limiting enzyme^33^. In *R. australe*, *SAM synthase2* (*SAM2*), *ACO1*, and *ACO5* transcripts showed up-regulated expression in LHA and L4. ET is considered an important plant stress hormone besides its role in growth and development, fruit ripening, senescence, submergence, pathogens, and response to gravity. ET is also reported to regulate salt and cold stress tolerance^34,35^. The ERF family of TFs performs diversified functions in terms of hormone response, development, and biotic and abiotic stress responses^36^. In *A. thaliana*, overexpression of several *ERF*s enhances tolerance to salt, drought, light-stress, cold-stress, heat, and pathogens^37^. In the present work, *ERF5*, *ERF010-like*, *ERF010*, and *ERF13* showed up-regulated expression in LHA and L4 which is in agreement with the positive correlation between ET biosynthesis and cold tolerance^38^. Moreover, enhanced expression of various defense-related genes was also observed in the present study which might be associated with the up-regulation of JA, SA, ET, and ERFs and could be linked with biotic and abiotic stress tolerance in *R. australe*.

Abscisic acid (ABA) is a stress hormone and its increased endogenous level leads to activation of many genes via ABA-responsive element binding factors (ABFs)^39^. β-Carotene hydroxylase (BCH) is associated with the biosynthesis of zeaxanthin, which is a carotenoid precursor of ABA. In rice, BCH mutants, *dsm2* showed a significant reduction in zeaxanthin and ABA content under drought stress^40^. ABA-dependent and ABA-independent pathways could interact and regulate various genes involved in cold and osmotic stress^41^. Also, the exogenous treatment of ABA at normal temperature has been found to enhance freezing tolerance^42^. In the present study, transcripts encoding *zeaxanthin epoxidase* (*ZEP*)*, BCH*, *phytoene synthase* (*PSY*), *lutein deficient5*/*CYP97A3*, *ABF*, and eight *protein phosphatases* (*PP2C*s) were up-regulated in LHA and L4. PP2Cs are controlled by ABA receptors which negatively regulate ABA responses. However, higher expression of *PP2C* was observed in *Populus euphratica* under freezing stress^42^. Therefore, ABA biosynthesis genes and PP2Cs might act in harmony to provide stress tolerance in *R. australe*.

Gibberellins (GAs) promote growth in various stages of the plant’s life. In the diterpenoid biosynthesis, gibberellic acid with its soluble receptor, GIBBERELLIN INSENSITIVE DWARF1 (GID1), forms GAs-GID1 complex which binds to DELLA protein. DELLA protein is a negative regulator of GA that degrades GA-GID1 complex via E3 ubiquitin ligase 26S proteasome pathway^43^. Gibberellin 3-oxidase1 (GA3ox1) is involved in the production of bioactive GAs. *GA3ox1* and *GA3ox2* affect vegetative development and are responsible for the synthesis of bioactive GAs in *A. thaliana*^44^. In the present work, *GID1B* was up-regulated, while *GA3ox1*, *gibberellin-regulated protein6* (*GRP6*), and *GRP13* showed down-regulation in LHA and L4. Hence, the possibility exists for the lower level of GA in *R. australe*. The reduction of GA has been reported in response to stresses such as osmotic, cold, and salt^43,45^. Moreover, ABA and GA have an antagonistic effect in various physiological processes^46^ and hence, a higher level of ABA and lower level of GA in the present work suggested a cross-talk in *R. australe* at 4 °C as well as in its natural habitat.

Among brassinosteroids (BRs), *somatic embryogenesis receptor kinase1-like* (*SERK1*) showed up-regulation, while *brassinosteroid insensitive1 kinase inhibitor1-like* (*BKI1*) was down-regulated in LHA and L4. SERK1 is a leucine-rich repeat transmembrane receptor-like kinase (LRR-RLK) and is known to be involved in the activation of brassinosteroid insensitive1 (BRI1) signaling pathway for BRs^47^. SERK1 was also induced by pathogen infection, SA, JA, and ABA. Overexpression of *SERK1* led to an increase in resistance to the fungus in rice^48^. BKI1 is a negative regulator of BR signaling and encodes a phosphoprotein that interacts directly with the kinase domain of BR receptor, BRI1^49^. BRI1 perceives the hormones and initiates signaling to control the activity of BR regulated TFs. BRs play very important roles in plant development and promote chilling and freezing tolerance^50^. Therefore, up-regulation of *SERK1* and down-regulation of *BKI1* could result in the enhancement of BRs in *R. australe* and might be involved in defense signaling and cold tolerance besides growth and development of plant.

Cytokinin dehydrogenase (CKX) is a key catabolizing enzyme and is involved in the irreversible degradation of CKs. In the present study, *CKX1* and *CKX7* transcripts were up-regulated in LHA and L4, resulting in the lower level of CKs in *R. australe*. Beside CKs degradation, CKX has been shown to enhance drought, salinity, and heat-stress tolerance along with plant development^51^. Hence, the low level of CKs in *R. australe* might be involved in stress tolerance and maintain the plant growth under adverse conditions in its niche and at low-temperature (4 °C).

Auxin is an important plant hormone that participates in several cellular and developmental processes. Auxin response factors (ARFs) are TFs that regulate (activate or repress) auxin-responsive gene expression by binding to the auxin-responsive elements (TGTCTC) which are found in the promoters of auxin-responsive genes^52^. In the present work, up-regulation of *ARF18*, *TRANSPORT INHIBITOR RESPONSE1* (*TIR*)*1* and down-regulation of *indole-3-acetic acid* (*IAA*)*27*, *AUXIN* (*AUX*)*15A* in both LHA and L4 suggested a higher level of auxin in *R. australe*. Global gene expression analysis of *A. thaliana* during cold acclimation revealed alteration in the expression of Aux/IAA and ARFs^53^ and up-regulation of IAA biosynthesis-related genes in rice under cold and heat-stress^54^. Auxin signaling, concentration, and distribution through polar transport were affected under abiotic stress^54^. The cell-to-cell active movement of auxin from aerial tissues is known as polar auxin transport (PAT)^55^. PIN-FORMED (PIN)5 is an auxin efflux carrier, though it does not have a direct role in PAT but regulates intracellular auxin homeostasis and metabolism. PIN5 is localized in the endoplasmic reticulum (ER) and transports auxin from cytosol to the ER lumen. PIN5 appears necessary for fine-tuning of auxin function, since minor defects were observed in the knock-out *pin5 A. thaliana* mutants^56^. WALLS ARE THIN1 (WAT1) encodes another class of auxin transporter across the tonoplast membranes in the vacuole and regulates auxin homeostasis^57^. Also, 5NG4, an auxin-induced putative transmembrane protein is induced by auxin and has a possible role in transport^58^. In the present work, *PIN5*, *WAT1*, and *5NG4* were up-regulated in LHA and L4, which suggested the importance of intracellular transport mechanism and compartmentalization in controlling auxin homeostasis.

These observations suggested modulation of endogenous levels of auxin through abiotic stresses. In *R. australe*, down-regulation of AUX/IAA transcripts might play a role in balancing the auxin pool and impart auxin-mediated stress tolerance at different temperatures. Therefore, these phytohormones could play an important role in the adaptation of *R. australe* in its niche location (Fig. 6).

Intense irradiance including UV rays can impact the plant’s performance at HA and cause various DNA aberrations^59^. Therefore, the plant needs a strong DNA-repair system that maintains genomic stability and sustains the integrity of an organism^59^. According to previous reports, DNA damage repair protein might impart tolerance to UV-B induced DNA damage in *V. vinifera*^60^. Similarly, UV-B receptor (UVR8) acts as a UV-B photoreceptor and provide UV-protective responses in association with plant circadian clock^61^. Early light-induced proteins (ELIPs) protect plant leaves from photooxidation and acts as a photoprotective protein under high-light^62^. In the present study, transcripts encoding *DNA-repair protein* (*RAD51*), *UV-stimulated scaffold proteinA, UVR8*, *ELIP*, and *telomere repeat-binding protein3* showed up-regulation in LHA and L4 (Fig. 6) and hence, might help *R. australe* to activate a strong protection system in its niche conditions.

Wounding can harm plants most and it can be caused by herbivory, parasitism, or by mechanical injury from wind, snow, and fire. Various wound-inducible defense-related proteins and metabolites such as pathogenesis-related (PR) proteins, jasmonates, ABA, and ET are produced either in the vicinity of the wounding site alone or in undamaged parts of the wounded plants as well^63^. In the present work, transcripts encoding for *chitinase*, *classIVchitinase* (*CHI4*), *PR proteins*, *thaumatin-like protein* (*TLP*), *wound-induced protein* (*WIN*), *disease resistance RPP13-like protein1*, and *callose synthase* (*CALS*) showed up-regulation in LHA and L4 (Fig. 6). Hence, the enhanced expression of various defense-related genes might suggest a cross-talk mechanism in biotic and abiotic stresses in *R. australe*.

Plants rearrange their metabolic activities to adapt to the changing environment. In *R. australe* “phenylpropanoid biosynthetic process” was significantly enriched. Various environmental stresses, such as pathogen attack, wounding, UV, high-light, nutrient deficiencies, high and low-temperature, and herbicide treatment lead to increase in the accumulation of phenylpropanoids in plants^64^. Phenylpropanoid pathway and its branches of secondary metabolites are essential for plant development. For example, lignin for mechanical support, flavones, and flavonols for UV protection, anthocyanins, chalcones, and aurones as pigments for the pollination and seed distribution; and isoflavonoids as phytoalexins for defense^65^. Similarly, GO term “caffeoyl-CoA O-methyltransferase activity” was found highly significant. Therefore, genes involved in lignin biosynthesis pathway were identified.

Lignin is a complex polymer of monolignols and strengthens cell wall to avoid cell collapse. Plants can alter their lignin content in response to various stresses^66^. Moreover, various lignin-synthesizing enzymes and biosynthesis genes are up-regulated in response to cold stress in many plants^30,66^. In *R. australe*, *phenylalanine ammonia-lyase* (*PAL*), *4-coumarate-CoA ligase* (*4CL*), *p-coumarate 3-hydroxylase* (*C3H*), *caffeic acid 3-O-methyltransferase* (*COMT*), *caffeoyl-CoA O-methyltransferase* (*CCoAOMT*), *caffeoyl shikimate esterase* (*CSE*), *cinnamyl-alcohol dehydrogenase* (*CAD*), and *CAD4* transcripts showed up-regulation in LHA and L4 (Fig. 6). Also, *PAL*, *COMT*, and *CCoAOMT* showed enhanced expression in response to cold as previously reported in *Phaseolus vulgaris*^22^. In *A. thaliana*, expression of *4CL* was enhanced during lignin deposition in cotyledons, stems, and roots^67^*.* CAD is a committed enzyme in the monolignol pathway of lignin biosynthesis and is also induced by infection in *A. thaliana* and rice^68^. *CAD* also showed enhanced expression in *Ipomoea batatas* or *A. thaliana* in response to cold^22^. PRXs, are known for lignin and suberin biosynthesis which could enhance cell wall rigidity by increasing cross-linking of cell wall components^24^. Up-regulation of various transcripts encoding *PRX* in the present study also indicated enhanced lignification mediated stress tolerance.

Flavonoids are a large group of polyphenolic secondary metabolites in plants^59^ and their accumulation during cold acclimation is reported in alpine and polar plants^69,70^. Chalcone synthase (CHS) is the first committed enzyme in flavonoid biosynthesis. In many plants, terpenoid biosynthetic genes showed enhancement after mechanical wounding^71^ and in the niche of *R. australe*, mechanical wounding is frequent due to extremes of environmental conditions such as snow, hailstorm, and high wind velocity. 3-Hydroxy-3-methylglutaryl coenzyme A reductase (HMGR) and sesquiterpene synthase are two very important genes of terpenoid biosynthetic pathway. In potato, HMGR was expressed in response to the pathogen, elicitor, and wounding^72^. Similarly, sesquiterpene synthase was up-regulated by SA in *Polygonum minus*^73^. Therefore, terpenoid pathway might play an important role in adaptation of *R. australe*.

Shikimate pathway is involved in the production of aromatic amino acids therefore, pathway gene, such as *chorismate mutase* (*CM*) is a potential target for herbicides, fungicides, and antibiotics^74^. In *R. australe*, *flavonol synthase/flavanone 3-hydroxylase* (*FLS*), *CHS2* of flavonoid pathway; *HMGR*, *sesquiterpene synthases* of terpenoid pathway; and *CM2*, *CM3* of shikimate pathway showed up-regulation in LHA and L4 as compared to that in L25 (Fig. 6).

In this study, transcripts related to metabolism and biosynthesis of lignin and terpenoid pathway showed up-regulation in LHA and L4 as compared to that in L25. All these genes were mapped through KEGG pathway analysis and are shown in Supplementary Figures S11-12. Collectively, data showed that above mentioned genes associated with secondary metabolism could enhance phenolic compounds, lignification, and would correlate with stress tolerance in its niche location and at 4 °C.

Sugar plays a pivotal role as a signaling molecule, osmoregulator, cryoprotectant, and is associated with the enhancement of multi-stress tolerance in plants^23^. Trehalose, a nonreducing disaccharide acts as a signaling molecule and serves as a stress protectant for proteins and cellular membranes against various environmental conditions^23^. Transgenic *A. thaliana* overexpressing *trehalose-phosphate synthase* (*TPS*)*1–TPS2* displayed significantly enhanced drought, freezing, and salt tolerance^75^. Raffinose is a major soluble carbohydrate in seeds, roots, and tubers. It acts as an osmoprotectant, antioxidant, and could serve as a signal in response to several abiotic or biotic stresses^76^. Galactinol-sucrose galactosyltransferase (RFS) enzyme belongs to the family of glycosyltransferases and is involved in the synthesis of raffinose. In *R. australe*, *TPS9*, *glucose 6-phosphate* (*G6P*) of trehalose synthesis and *RFS2-like protein*, *RFS6* of raffinose synthesis were up-regulated in LHA and L4. Hexokinase (HXK) is a sugar sensor and acts as a core component in plant sugar signaling pathways^77^. HXK1 integrates the nutrient and hormone signals to modulate gene expression in response to environmental cues in *A. thaliana*^78^. In cells, sucrose is hydrolyzed to hexoses by cell wall invertase (CWINV) or sucrose synthase (SUS) which increases the local hexose availability^79^. In the present study, *HXK2*, *CWINV*, and *SUS7* showed up-regulation. For sugar transportation, *SWEET1*, *sugar transporterERD6-like6, sugar transport protein* (*STP*)*6, STP7*, *and STP8* transcripts were up-regulated, while *SUC1* and *SUT1* transcripts were down-regulated in LHA and L4 as compared to that in L25 (Fig. 6).

HSPs (heat-shock proteins)/chaperones are considered as powerful buffers against environmental stress such as heat, cold, UV, drought, osmotic, salt, high-light, oxidative, and pathogen infection^80^. Also, various heat stress TFs (HSFs) were modulated by abiotic stresses, phytohormones, and different developmental events^81^. DnaJ/HSP40 are key partners for HSP70. Overexpression of *BIL2*, a mitochondrial DnaJ/HSP40 homolog confers tolerance against salt stress and strong light, while its mutants showed reduced photosynthetic efficacy in transgenic *A. thaliana*^82^. The late embryogenesis abundant (LEA) proteins play diverse roles as an antioxidant, metal ion binding, membrane and protein stabilizer, and are often induced by abiotic stresses^83^. In the present study, *DnaJ8*, *DnaJ11*, *DnaJ sub familyC17*, *HSFB3*, *LEA*, *LEA1*-*like*, *LEA5, 11kDaLEA, dehydrinb*, *chaperonin-like*, and *chaperonin60* were up-regulated in LHA and L4 as compared to that in L25 (Fig. 6). Therefore, abundance of sugar moieties, sugar sensors, and sugar transporters in the present study might indicate a role of sugar mediated signaling, osmoprotection, energy distribution. Up-regulation of HSPs, HSFs, and LEAs might impart protein stability and integrity to membranes under different stresses in *R. australe* in its niche location as well as at 4 °C.

Under cold stress, genes related to photosynthesis and photosystem have been found to exhibit lower expression^84,85^. Moreover, growth and developmental activities in plants are restricted at HA because of the low-temperature that might limit photosynthetic activity^86,87^. In *R. australe*, transcripts encoding *chlorophyll a/b-binding protein* (*LHCP*), *LHCP13*, *LHCP21*, *LHCP26*, *PSIIproteinI*, and *PSIIproteinM* showed down-regulation in LHA and L4 as compared to that in L25 (Fig. 6). Lower expression of these genes in LHA and L4 could be related to their lower rate of turn over rather than their effect on photosynthetic rate, which is a subject of further study. Various genes related to photosynthesis were found suppressed under cold-stress in *A. thaliana*^53^ and in *Hordeum vulgare*^88^.

Membrane transport systems play crucial roles in plant development and in maintaining cellular homeostasis under stresses through cell-to-cell and/or organ-to-organ communication by relocating various compounds such as phytohormones, sugars, amino acids, potassium, iron, nitrate, boron, and silicon^89^. Improvement in the plant membrane transport systems could be used to enhance productivity under adverse conditions due to their impact on whole plant physiology^90^. In *A. thaliana*, increased expression of multiple transporters and channel protein genes have been observed in response to various abiotic stresses^91^. In the present work, *pleiotropic drug resistance* (*PDR*)*, PDR1, multidrug resistance-associated protein* (*MRP*)*, ABCB25, ABCC4, multidrug and toxic compound extrusion* (*MATE*), and aquaporins such as *plasma membrane intrinsic protein* (*PIP*)*1*;*3-like*, *PIP2*;*1-like*, *nodulin 26-like intrinsic protein* (*NIP5*;*1*), and *NIP6*;*1* were up-regulated in LHA and L4 as compared to that in L25 (Fig. 6).

The ATP-binding cassette (ABC) transporter family is known as one of the largest protein super-families present in all living organisms^92^. PDR transporters of the ABCG subfamily are present in plants and fungi and are associated with the response to various biotic and abiotic stresses such as detoxification, transport of phytohormones, and secondary metabolites^93^. In *Zea mays*, *MRP3* of the ABCC subfamily was involved in the transportation of anthocyanin pigment into the vacuole^94^*.* MATE transporters are known to have a wide range of functions including anthocyanin pigment uptake, iron translocation, and aluminum resistance^95^. Aquaporins are extremely conserved membrane proteins that facilitate water transport across the plasma membrane along with glycerol, urea, CO_2_, NH_3_, metal ions, and ROS. In *A. thaliana, NIP5*;*1* and *NIP6*;*1* are involved in boron transport^96^, *PIP2*;*5* showed enhanced expression by cold treatment^96^ and in transgenic rice, *PIP1*;*3* exhibited an enhanced level of chilling tolerance^97^. Thus, up-regulation of transporters and aquaporins might be associated with stress tolerance, transport of plant secondary metabolites, hormones, water status, and general growth and development in *R. australe*.

*Rheum nobile*^59^ is also an alpine plant that belongs to genus of *R. australe*, and hence it might be interesting to compare the transcriptome of these two species. The comparison showed that a total of 3,894 transcripts of *R. australe* exhibited similarity with *R. nobile* at 80% coverage, which included transcripts for RNA methylation, response to cadmium ion, macro pinocytosis, response to salt stress and so on. However, a total of 17,409 transcripts, which were associated with regulation of transcription, macro pinocytosis, embryo development ending in seed dormancy, protein ubiquitination, response to abscisic acid stimulus, etc. showed no match with *R. nobile* transcriptome (Supplementary Table S10). This difference in transcriptome might be due to that these are different species, and also that the experimental conditions of these two species were very different.

## Conclusion

**Figure 7:**
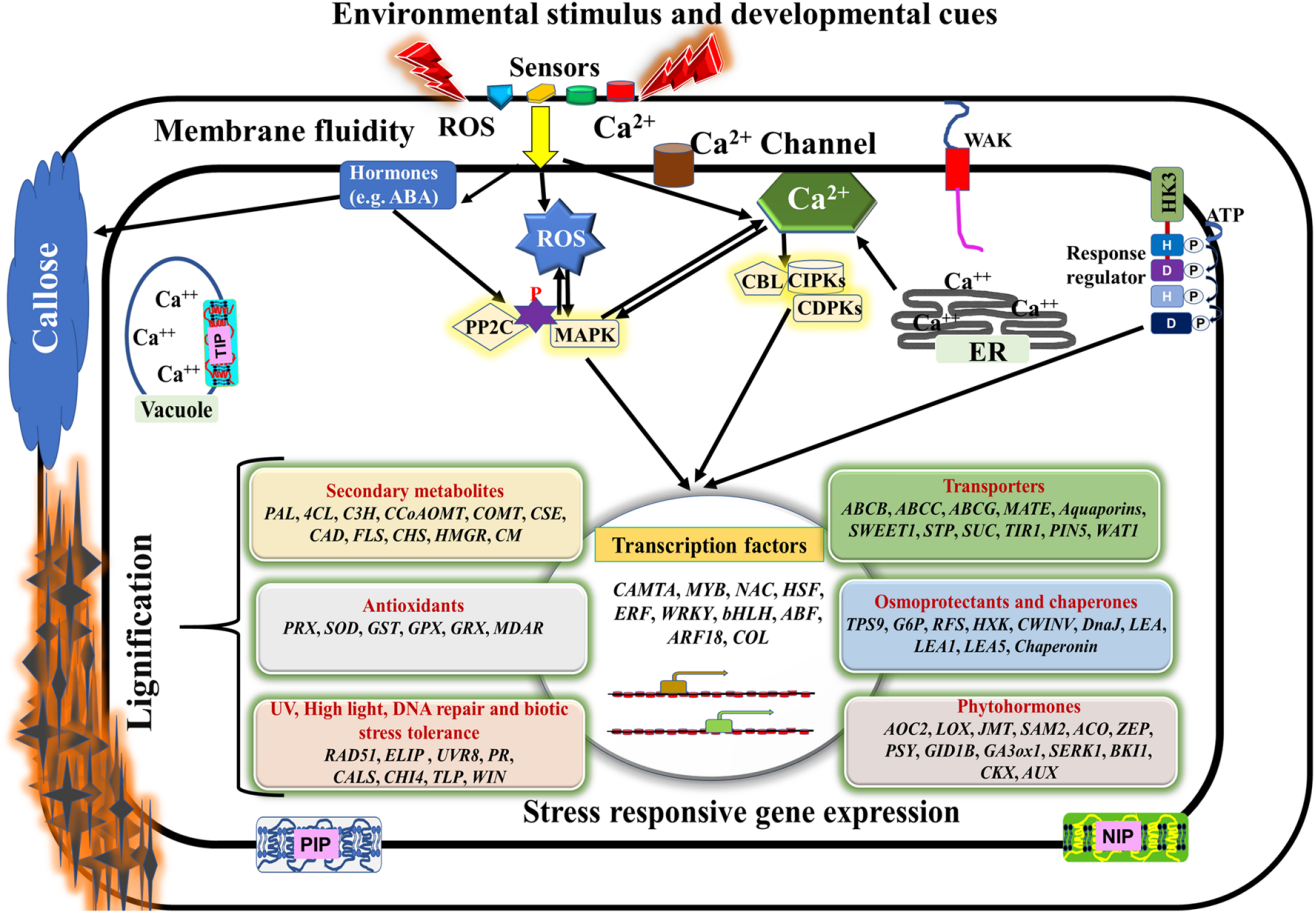
Proposed model depicting response of *Rheum australe* to environmental stresses. Environmental stress acts as a signal and are sensed by membrane-bound sensors such as plasma membrane proteins, HK, WAK and calcium channels. Sensors transmit the signal downstream which affects the homeostasis of chemical signals at the apoplastic space such as Ca^2+^, ROS, and alters membrane fluidity which stimulates a transient Ca^2+^ influx leading to activation of Ca^2+^, ROS, and hormone signaling cascades. These signaling molecules induce the activation of protein kinases (PKs) viz. CBLs, CIPKs, and CDPKs, and protein phosphatases (PPs) such as PP2Cs. MAPK cascades are centrally positioned in Ca^2+^ and ROS crosstalk as well as in the signal output after exposure to stress. Subsequently, these (PKs) and (PPs) deliver the information downstream and lead the activation of TFs through phosphorylation/dephosphorylation cascade. Plant hormone signaling is very important part of stress tolerance e.g. ABA signaling contributes to enhance callose deposition and modulates TFs. Activation of TFs and their binding to stress responsive gene promoters results in activation of stress responsive genes. The physiological changes that are manifested as membrane modification through increased lignification, metabolic enzymes, transporters, osmoprotectants, and DNA-repair system reprogram the cell and enable *R. australe* to achieve stress tolerance. Gene description and expression details are given in Supplementary Table S6. Abbreviations are given in the Supplementary Table S9. Genes involved in lignification are represented in bold text (Supplementary Table S9). The model has been drawn based on our data and the ideas to represent data has been taken from Kissoudis, C. et al., 2014; Fig. 1^119^ and Rejeb, I. et al., 2014; Fig. 2^120^.

The present study identified common genes which showed a similar pattern of expression in *R. austral*e in its natural habitat as well as in response to low-temperature (4 °C) as compared to that in at 25 °C. Among these, genes associated with Ca^2+^ signaling, protein kinases, and ROS might play an important role in quick sensing and signaling, while genes associated with various hormones would play a role in signaling and cross-talk between various stresses. Up-regulation of several antioxidants, chaperones, and osmoprotectants suggested protection of the cellular machinery, while the genes associated with mechanical wounding might be involved in offering protection against biotic stress. Also, genes associated with secondary metabolites, DNA-repair system against high-light, and UV could be involved in protection against multiple stresses. Data suggested the importance of plant signaling and protective mechanism in helping the species to adapt in the alpine environment of HA (Fig. 7).

## Methods

### Plant material, sample collection, and RNA isolation

For study of plants growing in its niche location, apical leaf from the top was harvested from the plants growing at Rohtang Pass (altitude ~ 4000 m amsl; 32° 22′ 19″ N; 77° 14′ 46″ E, Distt. Kullu, H.P.) in the month of August between 11:00 hrs-13:00 hrs (temperature, 9.36 ± 3 °C; light-intensity, 1642 *µ*molm^-2^ s^-1^; relative-humidity 74.2 ± 10%). For further studies, rhizomes of similar size of *R. australe* were collected from the same site^98^ and grown in pots maintained in a greenhouse (temperature, 25 ± 3 °C; light-intensity, 500–800 *µ*molm^-2^ s^-1^; relative-humidity 60 ± 10%) at our institute at Palampur (~ 1300 m amsl; 32° 06′ 32″ N; 76° 33′ 43″ E, Distt. Kangra, H.P.). After two months, plants were shifted to two separate plant growth chambers (light-intensity, 200 *µ*molm^-2^ s^-1^; relative-humidity 70 ± 10%; light/dark cycles, 12/12 h) maintained at 4 °C and 25 °C (Supplementary Fig. S1a-f). Apical leaves from the top of five different plants, grown from different rhizome were collected after one month between 11:00 hrs-13:00 hrs and pooled to form a biological replicate, frozen immediately in liquid nitrogen and stored at −80 ℃. Total RNA was extracted as described previously^99^ and quantified using a nanodrop 1000 (NanoDrop Technologies, USA). RNA integrity was checked on 1% denaturing formaldehyde agarose gel and Agilent 2100 Bioanalyzer (Agilent Technologies, USA).

### Library construction and RNA sequencing

Three RNA-Seq libraries were prepared from mRNA, purified from 5 μg of total RNA using TruSeq RNA sample preparation Kit v2 (Illumina Inc., USA) as per the manufacturer’s instructions. Quantification of PCR enriched libraries was performed on fluorescence-based assay on a fluorometer using Qubit dsDNA BR assay kit (Life Technologies, USA). Average insert size of the library was of 250 bp and was checked using DNA1000 kit (Agilent Technologies, USA) on an Agilent 2100 Bioanalyzer. A total of 10 pM of each library (representing one sample) was used to generate clonal clusters in the flow cell using TruSeq PE Cluster Kit v5 on a cluster station (Illumina Inc., USA). PE 2 × 72 bp sequencing was performed on amplified clusters using Illumina Genome Analyzer IIx (Illumina, USA) as per the manufacturer’s instructions.

### De novo assembly, functional domain search, read mapping, and transcript abundance measurement

In house developed tool-filteR was used to filter out poor quality reads, read trimming as well as for adapter removal as described previously^100^. De novo assembly of high-quality reads was done using SOAPdenovo-Trans assembler^101^. Evaluation of the assembly quality was done by calculating and optimizing k-mer size, N50 value, coverage, and the average length of the assembled transcript sequences. The sequence redundancy was removed with hierarchical clustering by subjecting the sequences to CD-HIT-EST^102^ at 90% similarity along with TGICL-CAP3^103^ clustering tool, followed by homology search against NR protein database using BLASTX with E-value of 10^–5^ to identify unigenes^104^. DS clustering was performed to assign the same group to the contigs/scaffolds and to cluster the assembled transcript sequences into single unigene. No hit contigs/scaffolds were searched against the CDD^105^ using RPS-BLAST with E-value of 10^–5^. Clean reads were mapped back to the assembled transcripts using Bowtie2^106^ to estimate total mapped reads with the allowance of two mismatches and assigned to unigenes using the RSEM software^107^. The expression level of all the unigenes were calculated with the FPKM mapped read method.

### Functional annotation, data analysis of unigenes, and DEGs

Assembled unigenes of *R. australe* were searched against the GO database, KEGG, and EC number using annot8r^108^ tool with E-value of 10^−1^. WEGO annotation plot^109^ was used for GO functional classification for a better understanding of gene functions at the macro level. COG analysis was done for functional evaluation of assembled unigenes using BLASTX (E-value of 10^−5^)^110^. All the assembled unigenes were searched against the plant TFs database (PlantTFDB version 4) using BLASTX with E-value of 10^−5^. GC content analysis was done using “Emboss GeeCee” tool (http://emboss.bioinformatics.nl/cgi-bin/emboss/geecee)^111^. Significant DEGs with p-value ≤ 0.05, FDR ≤ 0.05, and log_2_ FC ≥ 2 were identified using edgeR package^112^ and the expression level was determined with the FPKM method. The DEGs were subjected to GO and KEGG enrichment analysis using AgriGO^113^ and KAAS^114^, respectively. Transcripts were annotated by "KEGG Mapper” (https://www.kegg.jp/kegg/tool/map_pathway.html) tool^115,116^. Permission to use KEGG pathway map images was kindly granted by “Junko Takigawa, Kanehisa Laboratories” in response to Ref: 200649, Dated: 12 October, 2020. The heat maps for DEGs were constructed using the R package version vol. 2 gplot^117^.

### Assembly validation by similarity search against the ESTs and expression validation of DEGs by qRT-PCR

To validate assembled transcripts, ESTs of *R. australe* were downloaded from the NCBI dbEST and BLASTN analysis was performed against *R. australe* transcriptome with E-value of 10^−5^. Also, to check the reliability and validation of expression level as obtained by RNA-Seq data, relative expression of 28 selected genes was measured using qRT-PCR. First-strand cDNA was synthesized using 1 μg of DNaseI-treated total RNA using a high capacity cDNA reverse transcription kit (Invitrogen, USA) following the manufacturer’s instructions. Primers for qRT-PCR analysis were designed using Primer Express software version 3.0.1 (Applied Biosystems, USA). The qRT-PCR was performed on a StepOne plus RT-PCR machine (Applied Biosystems, USA). Each reaction contained 1:10 diluted 2.5 μL cDNA, 10 mM each of forward and reverse gene-specific primer, and 5 μL SYBR Green qPCR Master Mix (Agilent Technologies, USA) in a final volume of 10 μL. qRT-PCR was performed with three technical and three biological replicates. Thermal cycling program used was as follows: 4 min at 94 °C, 40 times cycling for 30 s at 94 °C, 30 s at 51–60 °C, and 72 °C for 30 s with a final melting curve analysis. Relative fold change in the expression of target genes was calculated by using 2−^ΔΔCT^ method with *glyceraldehyde-3-phosphate dehydrogenase* (*GAPDH*) of *R. australe* as an internal control for qRT-PCR data normalization^118^.

### Ethics approval and consent to participate

This study including sample collection was conducted according to India’s Biological Diversity Act 2002 which permits use of biological resources to bonafide Indians for scientific research purpose^98^.

## Supplementary Information

Supplementary Information 1.

Supplementary Information 1.

Supplementary Table 1.

Supplementary Table 2.

Supplementary Table 3.

Supplementary Table 4.

Supplementary Table 5.

Supplementary Table 6.

Supplementary Table 7.

Supplementary Table 10.

## Data Availability

All data generated or analyzed in this study is included in the article and in its additional files. Illumina raw sequencing data of assembled contigs are submitted as BioProject PRJNA475866 to National Centre for Biotechnology Information (NCBI) in Sequence Read Archive (SRA).
