# Supplementary material for: Comparative transcriptome analysis of Rheum australe, an endangered medicinal herb, growing in its natural habitat and those grown in controlled growth chambers
Source: Sci Rep. 2021 Feb 12;11:3702. doi: 10.1038/s41598-020-79020-8 (PMC7881009; doi:10.1038/s41598-020-79020-8)
Supplement: Supplementary file 1 — Supplementary Information 1. [file 41598_2020_79020_MOESM1_ESM.docx]

**Supporting Information-I**

**Comparative transcriptome analysis of *Rheum australe*, an endangered medicinal herb, growing in its natural habitat and those grown in controlled growth chambers**

Deep Mala^1,2†^, Supriya Awasthi^3†^, Nitesh Kumar Sharma^2,3^, Mohit Kumar Swarnkar^1^, Ravi Shankar^2,3*^, Sanjay Kumar^1,2*^

**Affiliations**

1. Biotechnology Division, Council of Scientific and Industrial Research-Institute of Himalayan Bioresource Technology, P.O. Box 6, Palampur (H.P.)-176061, India.
2. Academy of Scientific and Innovative Research (AcSIR), Ghaziabad-201001, India.
3. Studio of Computational Biology & Bioinformatics, Biotechnology Division, CSIR-Institute of Himalayan Bioresource Technology, Palampur-176061 (H.P.), India.

† Equal contributors

* Corresponding authors

**Email:** [deepmala.nine@gmail.com](mailto:deepmala.nine@gmail.com), [supriya.awasthy@gmail.com](mailto:supriya.awasthy@gmail.com), [prince26121991@gmail.com](mailto:prince26121991@gmail.com), [mohitswarnkar@gmail.com](mailto:mohitswarnkar@gmail.com), [ravish@ihbt.res.in](mailto:ravish@ihbt.res.in), [sanjaykumar@ihbt.res.in](mailto:sanjaykumar@ihbt.res.in)


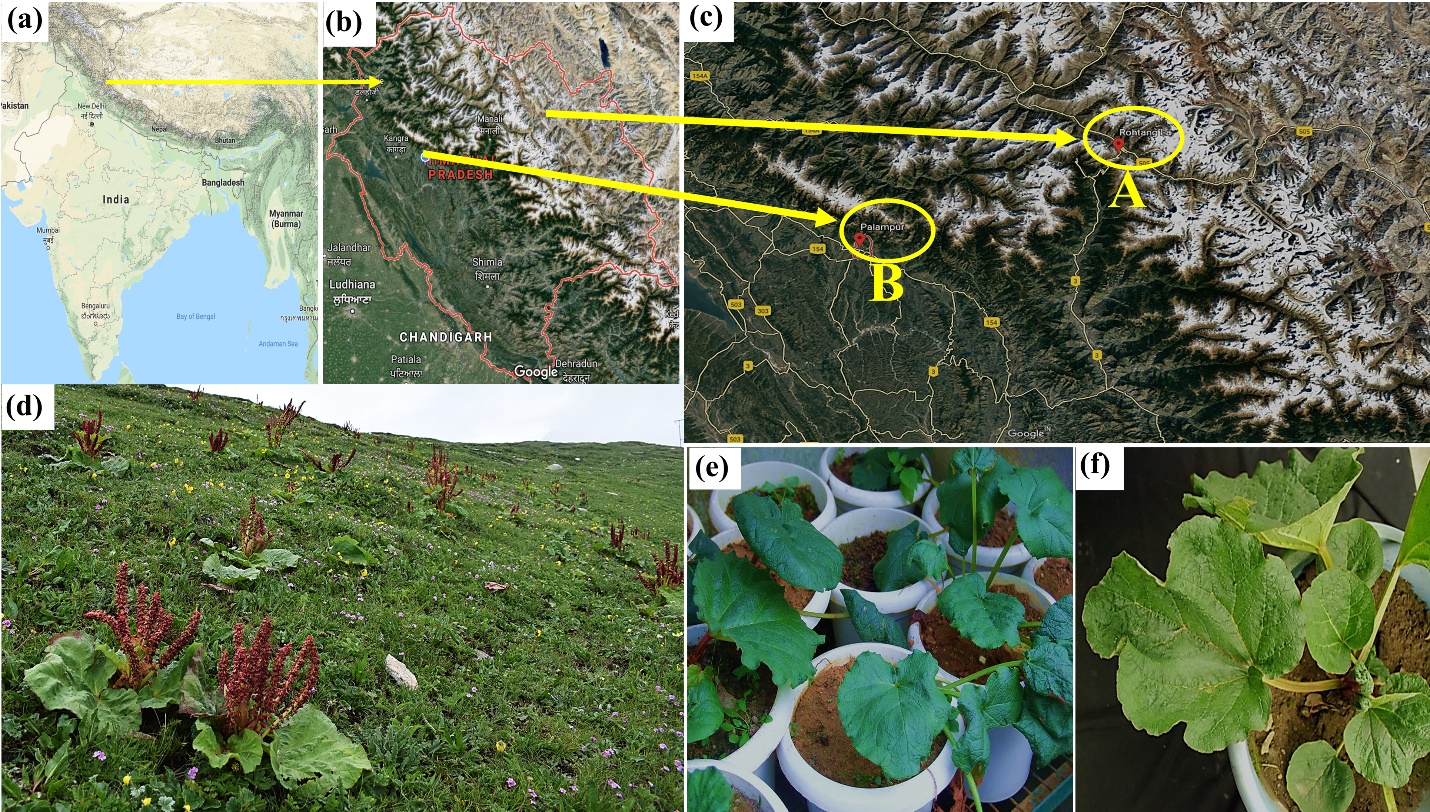


**Supplementary Figure S1. Study areas of *Rheum australe*.** (**a-c)** Locations and satellite map depicting the areas of collection of *R*. *australe* in Himachal Pradesh, India (retrieved from Google Earth). **(c)** Caption “A” represents “Rohtang Pass” and Caption “B” represents “Palampur”. **(d)** *R. australe* grown in the natural habitat of high altitude at Rohtang Pass, Himachal Pradesh. **(e)** *R. australe* grown in the polyhouse at Palampur, Himachal Pradesh. **(f)** *R. australe* grown in growth chamber at Palampur, Himachal Pradesh. Photographs **(d, e, f)** have been taken by our group.


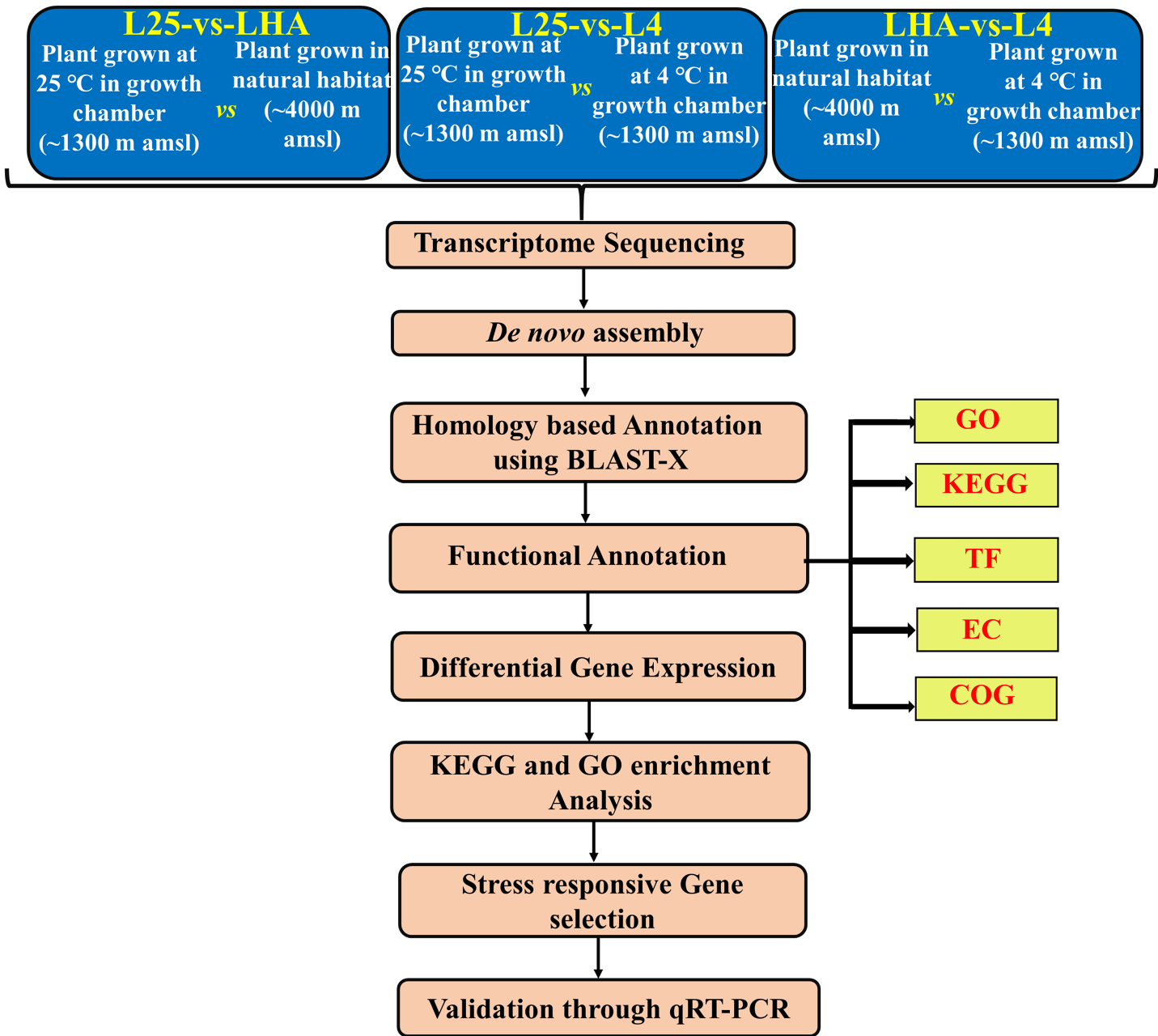


**Supplementary Figure S2. Summary of different analysis conditions, *de novo* assembly, and annotation details of the transcriptome of *Rheum australe*.** Leaf samples were collected from plants grown in the natural habitat of high altitude (LHA) and under controlled conditions in growth chambers at 25 °C (L25), and 4 °C (L4).


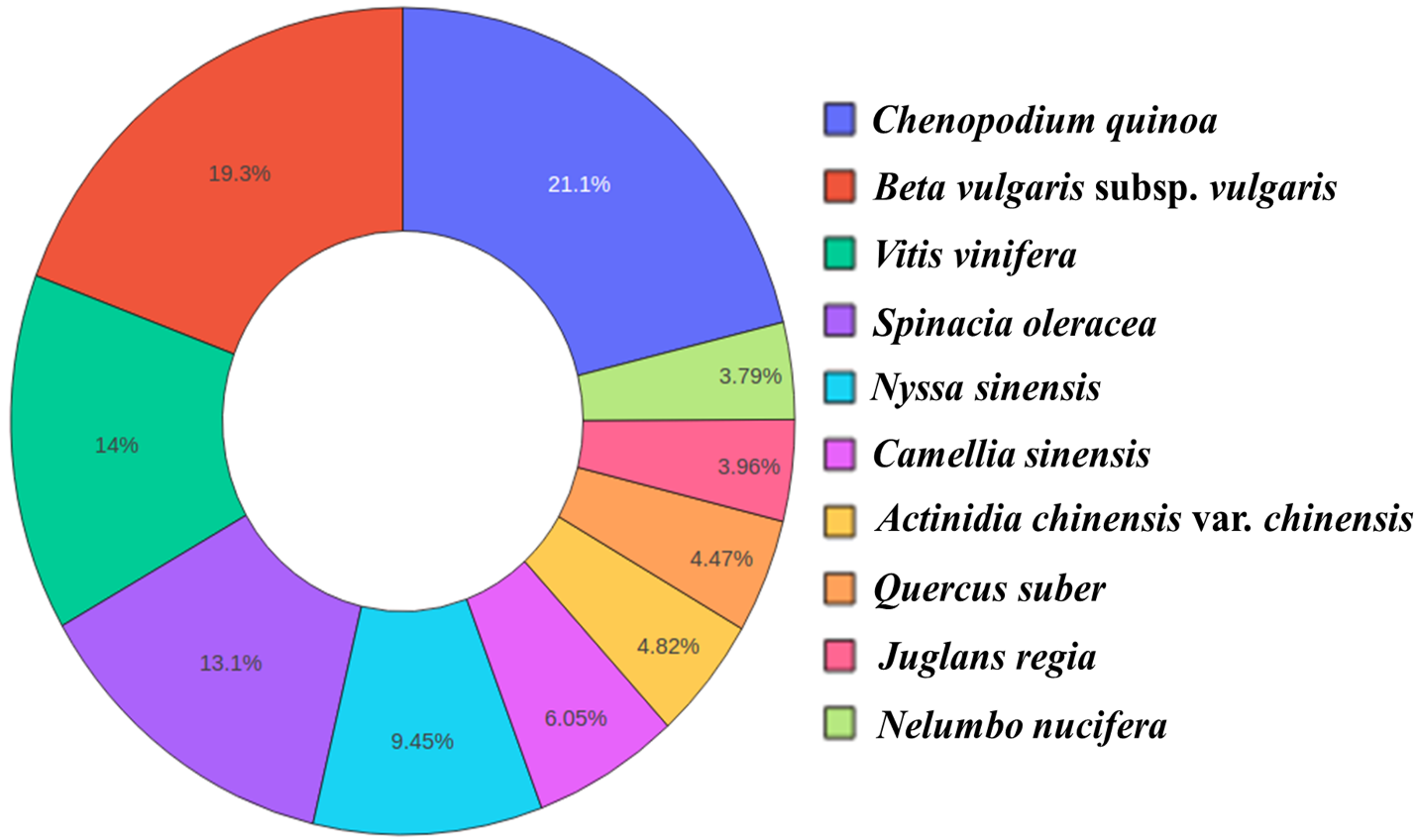


**Supplementary Figure S3.** **Species distribution of the BLASTX results of *Rheum australe* transcriptome.** The numbers in the pies indicate the percentage of unique reads in each category.


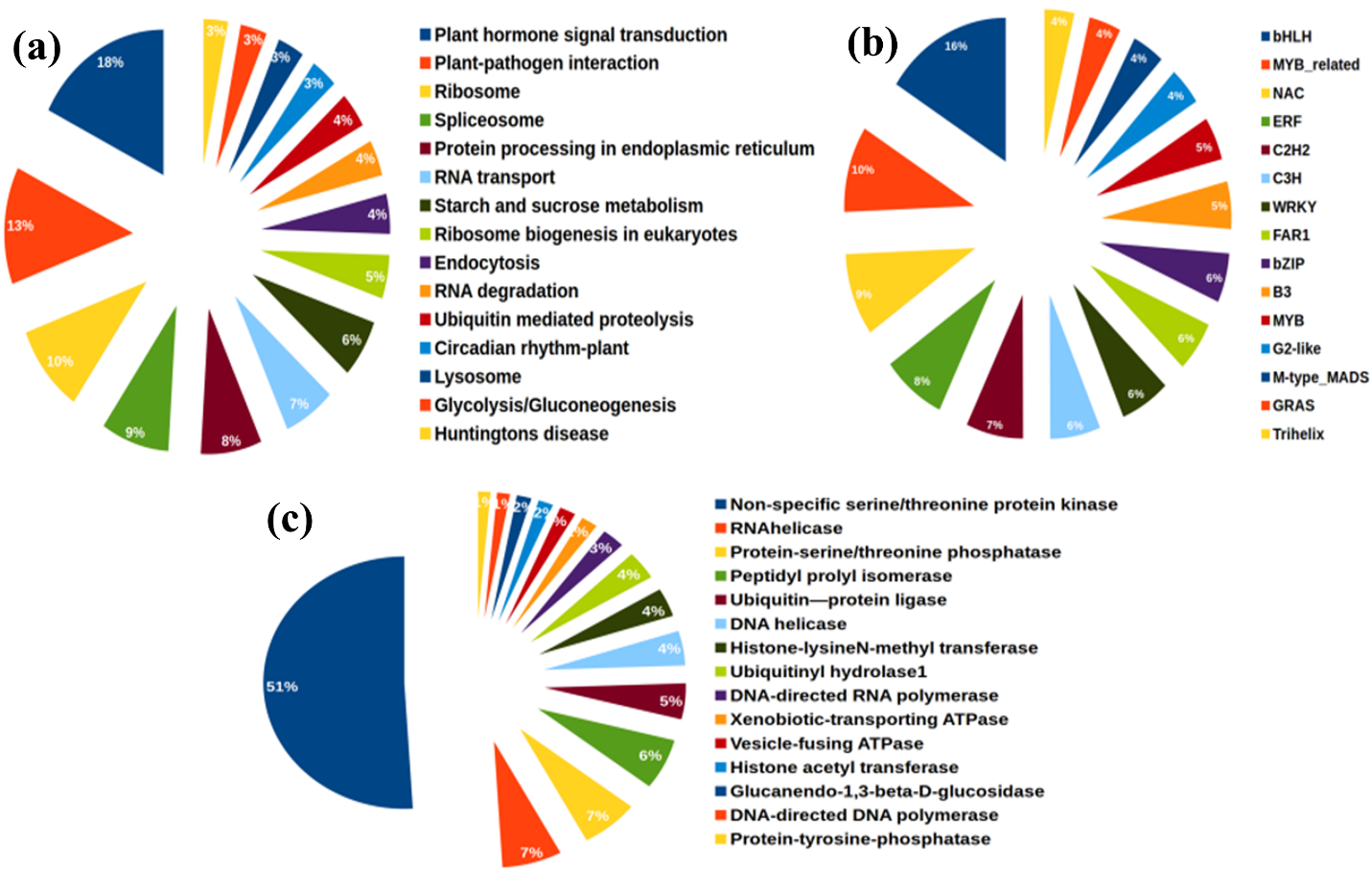


**Supplementary Figure S4.** Identification of top 15 unigenes by **(a)** “Kyoto Encyclopedia of Genes and Genomes” (KEGG), **(b)** transcription factor (TF) families by “The Plant TF database”, and **(c)** enzyme classes by “Enzyme Commission” (EC) classification. The area under each pie diagram represents the value in percentage.


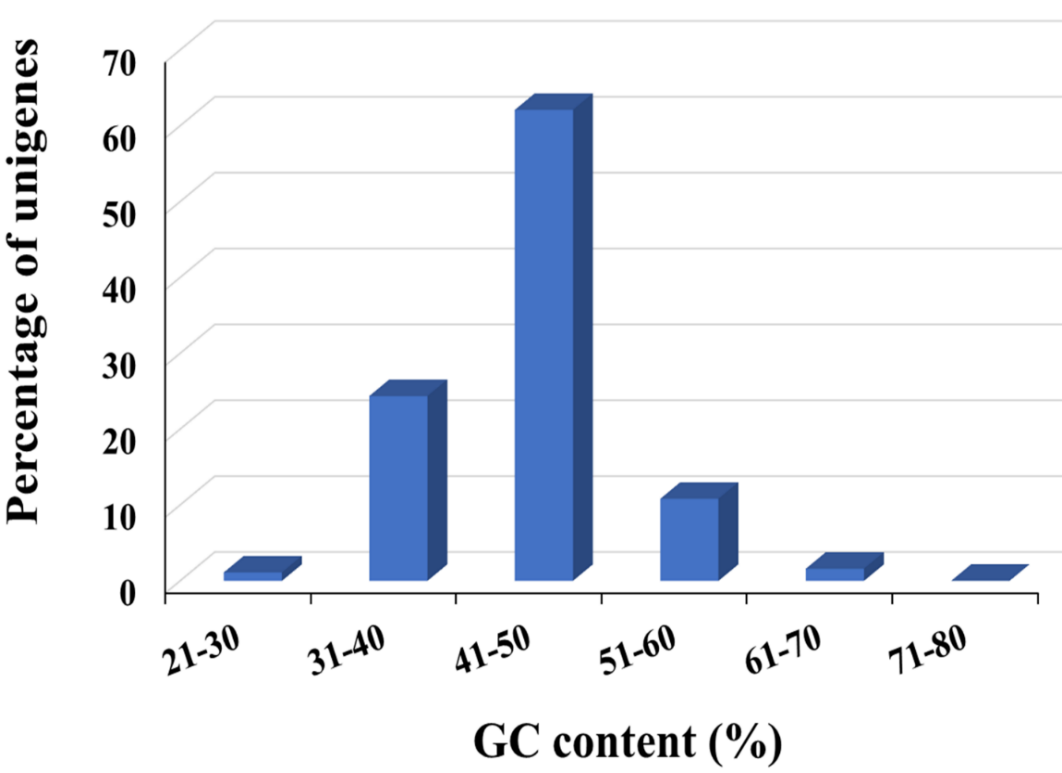


**Supplementary Figure S5. Percentage of Guanine-Cytosine (GC) content in *Rheum australe*.** X-axis indicates the percentage of transcripts and y-axis indicates the data range of GC content of unigenes.


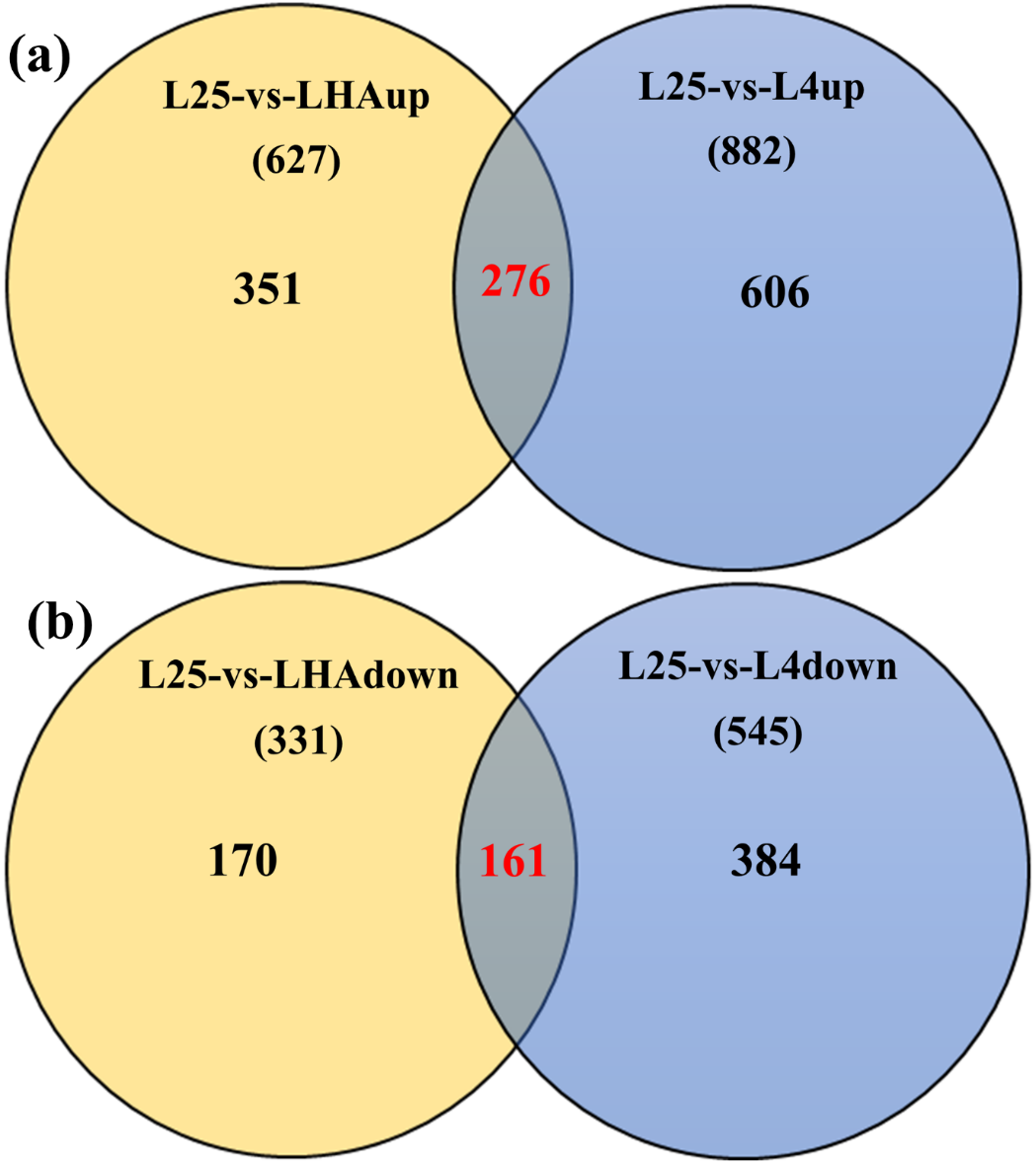


**Supplementary Figure S6. Venn diagram showing common up- and down-regulated differentially expressed genes (DEGs).** Up-regulation of 276 and down-regulation of 161 common DEGs in LHA and L4 as compared to that in L25.


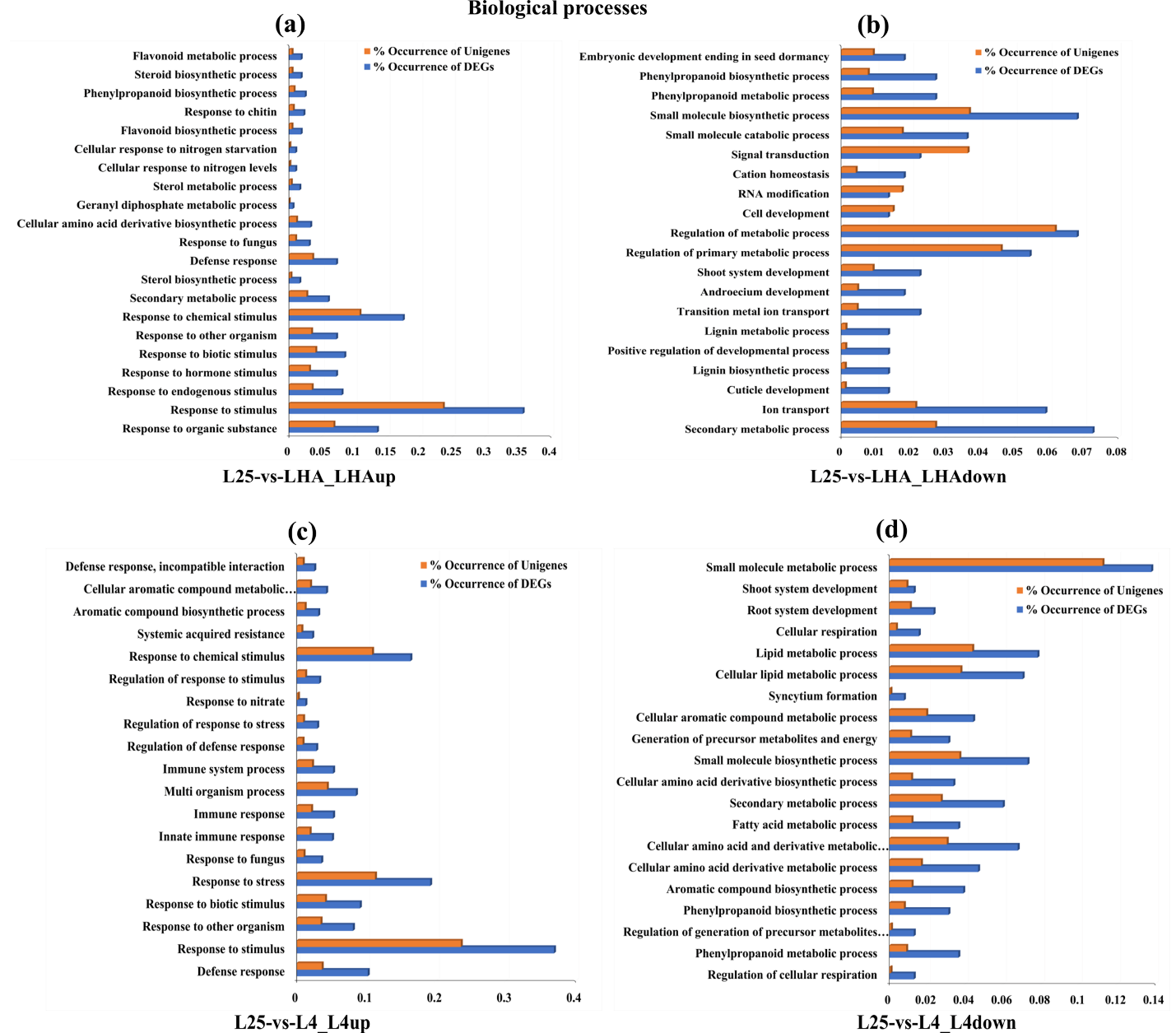


**Supplementary Figure S7: Classification of differentially expressed genes (DEGs) and unigenes under Gene Ontology (GO).** Top 20 DEGs and unigenes in terms of GO under biological processes category show **(a, c)** up-regulated and **(b, d)** down-regulated genes in L25-vs-LHA and L25-vs-L4, respectively. X-axis indicates the percentage and y-axis indicates the different processes.


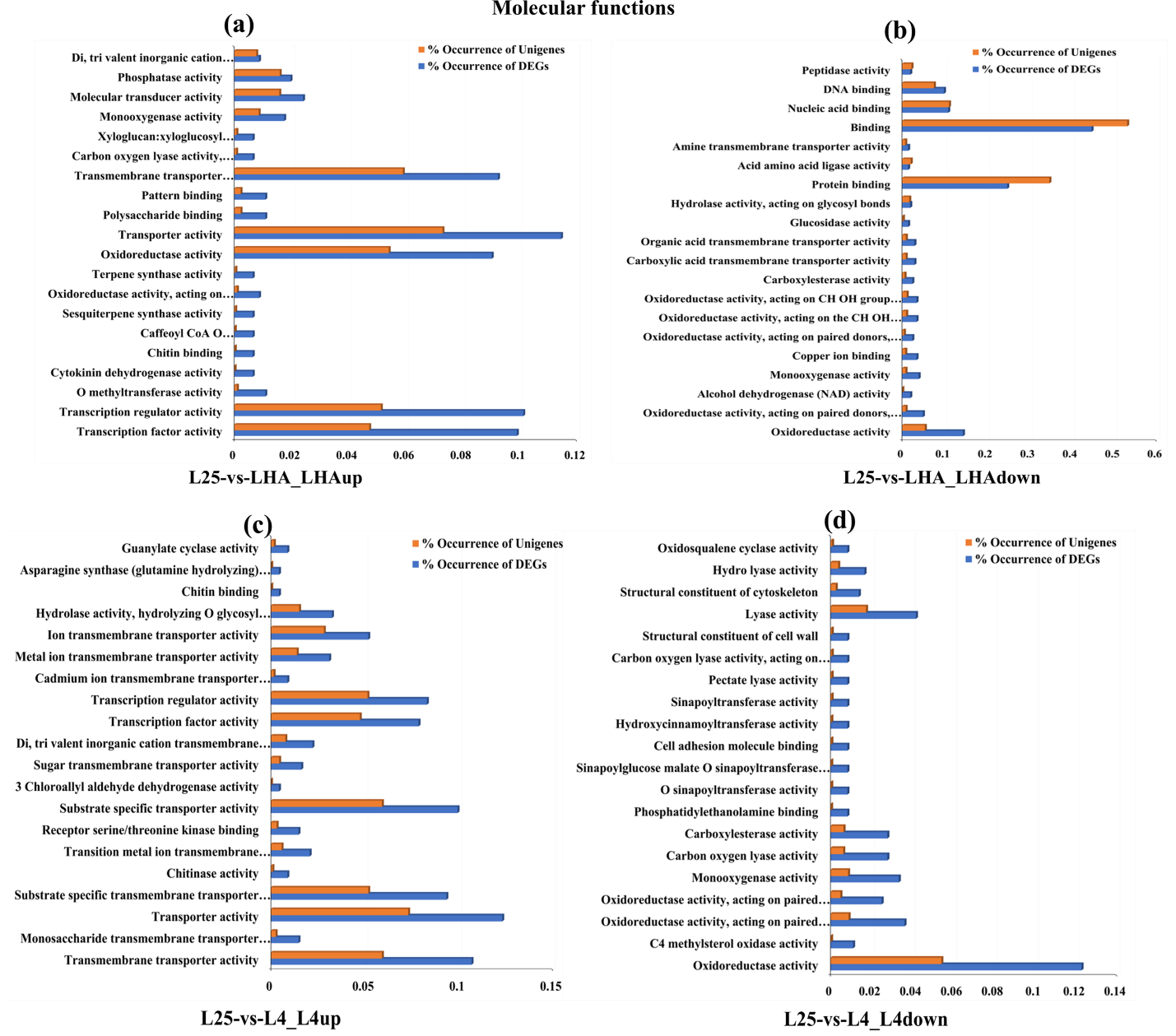


**Supplementary Figure S8.** **Classification of differentially expressed genes (DEGs) and unigenes under Gene Ontology (GO).** Top 20 DEGs and unigenes in GO term under molecular functions category show **(a, c)** up-regulated and **(b, d)** down-regulated genes in L25-vs-LHA and L25-vs-L4, respectively. X-axis indicates the percentage and y-axis indicates the different processes.


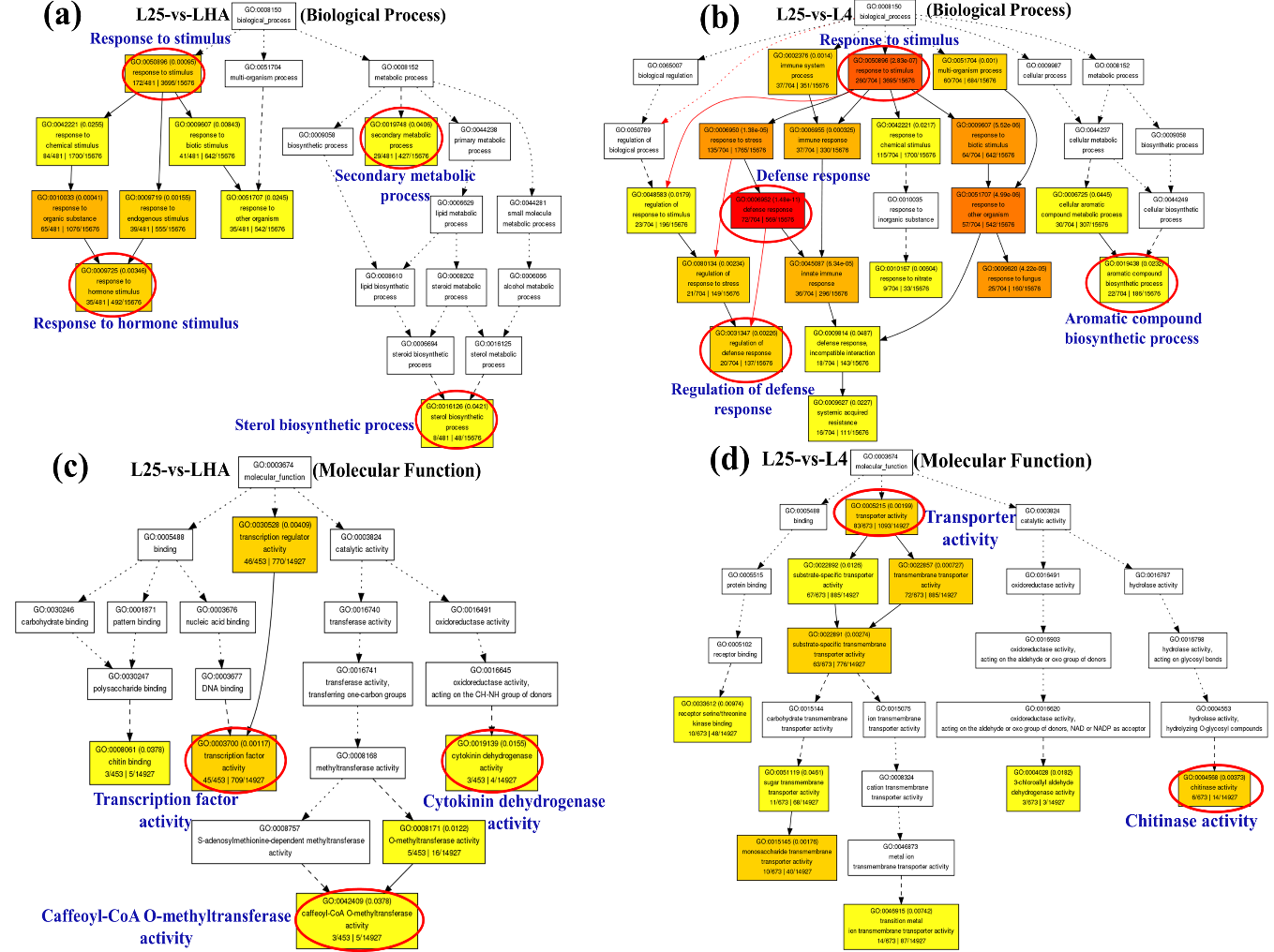


**Supplementary Figure S9.** **Gene Ontology (GO) enrichment analysis of differentially expressed genes (DEGs).** Hierarchical graph of DEGs in GO terms under (**a, b**) biological process and (**c,** **d**) molecular function category of upregulated DEGs in L25-vs-LHA and L25-vs-L4, respectively. Boxes in the graph represent GO terms with their GO ID, term definition and statistical information. Significant GO terms (p ≤ 0.05) are marked with color and non-significant GO terms are shown in white boxes. The degree of color saturation of a box is positively correlated to the significant level of the term.


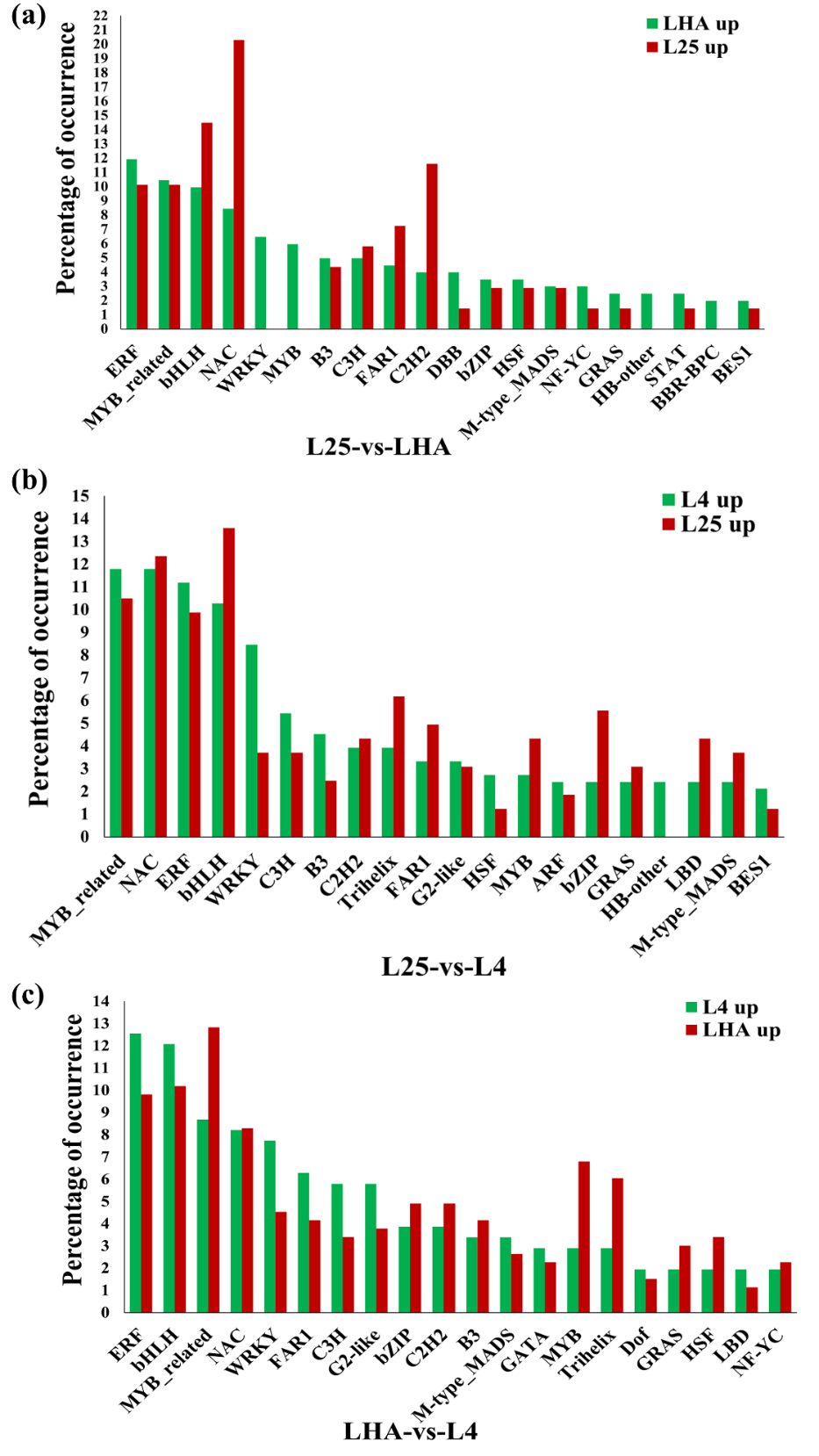


**Supplementary Figure S10. Identification of top 20 up-regulated transcription factors in L25-vs-LHA, L25-vs-L4, and LHA-vs-L4.** X-axis indicates the comparison conditions of DEGs and y-axis indicates the percentage of occurrence.

**Supplementary Table S8:** Number of up- and down-regulated transcription factors in L25-vs-LHA, L25-vs-L4, and LHA-vs-L4

|  | **DEGs** | **Up-regulated DEGs** | **Down-regulated DEGs** |
| --- | --- | --- | --- |
| **L25-vs-LHA** | 358 | 247 | 111 |
| **L25-vs-L4** | 632 | 415 | 217 |
| **LHA-vs-L4** | 602 | 251 | 351 |

**Supplementary Table S9:** Expanded form of abbreviations used in Figure 7. Genes involved in lignification are represented in bold text.

|  | **Abbreviations** |
| --- | --- |
| Figure 7 | **4CL: 4-coumarate CoA ligase**; ABA: Abscisic acid; **ABC: ATP-binding cassette**; ABFs: ABA-responsive element binding factors; ACO: 1-amino-cyclopropane-1-carboxylate oxidase; AOC: Allene oxide cyclase; ARF: auxin response factor; AUX: Auxin; BKI1: Brassinosteroid Insensitive1 Kinase Inhibitor1; bHLH: Basic helix-loop-helix; **C3H: p-Coumarate 3-hydroxylase**; **CAD: Cinnamyl alcohol dehydrogenase**; CALS: Callose synthase; CAMTA: Calmodulin-binding transcription activator; CBL: Calcineurin B-like proteins; **CCoAOMT: Caffeoyl-CoA O-methyltransferase**; CDPK: Calcium-dependent protein kinases; CHI4: class IVchitinase; **CHS: Chalcone synthase**; CIPK: CBL-interacting protein kinases; CKX: Cytokinin dehydrogenase; **CM: Chorismate mutase**; COL: CONSTANS-Like; **COMT: Caffeic acid 3-O-methyltransferase**; **CSE: Caffeoyl shikimate esterase**; CWINV: Cell wall invertase; ELIPs: Early light-induced proteins; ER: Endoplasmic reticulum; ERF: Ethylene response factor; FLS: Flavonol synthase/flavanone 3-hydroxylase; G6P: Glucose-6-phosphate; GA3ox: Gibberellin 3-oxidase; GID: GA INSENSITIVE DWARF; GPX: Glutathione peroxidase; GRX: Glutaredoxin; GST: Glutathione S transferase; HK: Histidine kinase; HMGR: 3-hydroxy-3-methylglutaryl-coenzyme A reductase; **HSF: Heat stress transcription factor**; HXK: Hexokinase; JMT: Jasmonate O-methyltransferase; LEA: Late embryogenesis abundant; LOX: Lipoxygenase; MAPK: Mitogen-activated protein kinase; MATE: Multidrug and toxic compound extrusion; MDAR: Monodehydroascorbate reductase; **MYB: Myeloblastosis**; NAC: NAM- ATAF1,2-CUC2; NIP: Nodulin-26 like intrinsic protein; **PAL: Phenylalanine ammonia-lyase**; PIN: PIN-FORMED; PIP: Plasma membrane intrinsic protein; PP2C: Protein phosphatases; PR: Pathogenesis related; **PRX: Peroxidase**; PSY: Phytoene synthase; RAD51: DNA-repair protein; RFS: Galactinol-sucrose galactosyltransferase; ROS: Reactive oxygen species; SAM2: S-adenosylmethionine synthase; SERK: Somatic embryogenesis receptor kinase; **SOD: Superoxide dismutase**; STP: Sugar transport protein; SUS: Sucrose synthase; TIR: TRANSPORT INHIBITOR RESPONSE; TLP: Thaumatin-like protein; TPS: Trehalose phosphate synthase; UV: Ultraviolet; UVR: UV-B receptor; WAK: Wall-associated receptor kinases; WAT: Walls are thin; WIN: Wound-induced protein; WRKY: WRKY transcription factor; ZEP: Zeaxanthin epoxidase |
