# Supplementary material for: Comparative transcriptome analysis of Rheum australe, an endangered medicinal herb, growing in its natural habitat and those grown in controlled growth chambers
Source: Sci Rep. 2021 Feb 12;11:3702. doi: 10.1038/s41598-020-79020-8 (PMC7881009; doi:10.1038/s41598-020-79020-8)
Supplement: Supplementary file 2 — Supplementary Information 1. [file 41598_2020_79020_MOESM2_ESM.docx]

**Supporting Information-II**

**Comparative transcriptome analysis of *Rheum australe*, an endangered medicinal herb, growing in its natural habitat and those grown in controlled growth chambers**

Deep Mala^1,2†^, Supriya Awasthi^3†^, Nitesh Kumar Sharma^2,3^, Mohit Kumar Swarnkar^1^, Ravi Shankar^2,3*^, Sanjay Kumar^1,2*^

**Affiliations**

1. Biotechnology Division, Council of Scientific and Industrial Research-Institute of Himalayan Bioresource Technology, P.O. Box 6, Palampur (H.P.)-176061, India.
2. Academy of Scientific and Innovative Research (AcSIR), Ghaziabad, Uttar Pradesh-201002, India.
3. Studio of Computational Biology & Bioinformatics, Biotechnology Division, CSIR-Institute of Himalayan Bioresource Technology, Palampur (H.P.)-176061, India.

† Equal contributors

* Corresponding authors

**Email:** [deepmala.nine@gmail.com](mailto:deepmala.nine@gmail.com), [supriya.awasthy@gmail.com](mailto:supriya.awasthy@gmail.com), [prince26121991@gmail.com](mailto:prince26121991@gmail.com), [mohitswarnkar@gmail.com](mailto:mohitswarnkar@gmail.com), [ravish@ihbt.res.in](mailto:ravish@ihbt.res.in), [sanjaykumar@ihbt.res.in](mailto:sanjaykumar@ihbt.res.in)

**Supplementary method:**

Genes involved in lignin biosynthetic pathway and terpenoid pathway play important role in plant in response to various stresses^1-3^. In the present study, transcripts related to lignin and terpenoid pathway showed up-regulation in LHA and L4 as compared to that in L25. All these genes were mapped through KEGG pathway analysis using "KEGG Mapper” (https://www.kegg.jp/kegg/tool/map_pathway.html) tool and are shown in Supplementary Figure S11-12^4,5^. Permission to use KEGG pathway map images was kindly granted by “Junko Takigawa, Kanehisa Laboratories” in response to Ref: 200649, Dated: 12 October 2020.

**References:**

1. Dixon, R. & Paiva, N. Stress-Induced Phenylpropanoid Metabolism. *Plant Cell*. **7**, 1085–1097 (1995).
2. Moura, J. C. M. S., Bonine, C. A. V., de Oliveira Fernandes Viana, J., Dornelas, M. C. & Mazzafera, P. Abiotic and biotic stresses and changes in the lignin content and composition in plants. *J Integr Plant Biol*. **52**, 360–376 (2010).
3. Steele, C. L., Katoh, S., Bohlmann, J., & Croteau, R. Regulation of oleoresinosis in grand fir (*Abies grandis*): differential transcriptional control of monoterpene, sesquiterpene, and diterpene synthase genes in response to wounding. *Plant physiol*. **116**, 1497-1504 (1998).
4. Kanehisa, M. & Goto, S. KEGG: Kyoto Encyclopedia of Genes and Genomes. Nucleic Acids Res **28**, 27–30 (2000).
5. Kanehisa, M., Sato, Y., Furumichi, M., Morishima, K. & Tanabe, M. New approach for understanding genome variations in KEGG. Nucleic Acids Res **47**, D590–D595 (2019).


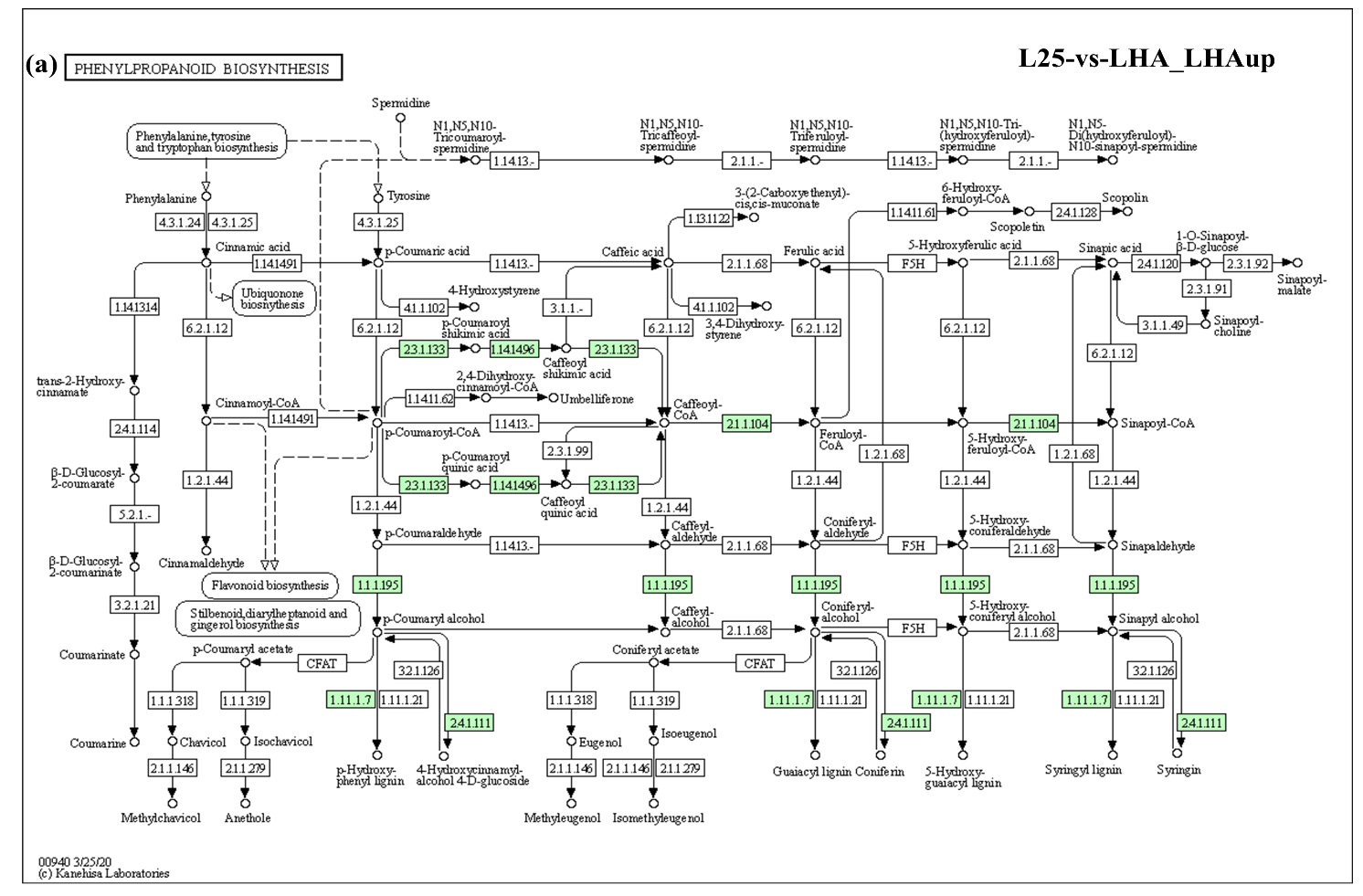


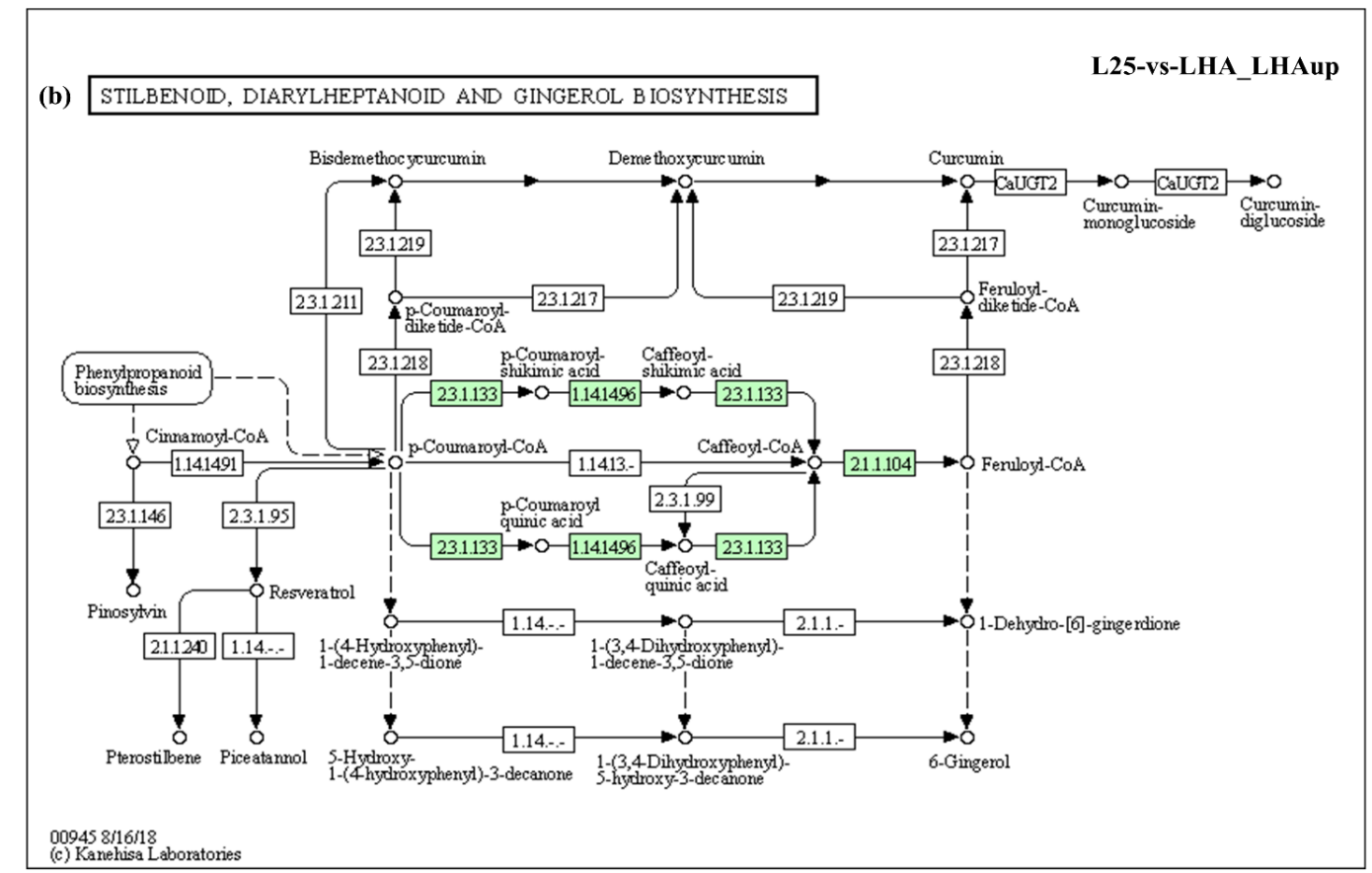


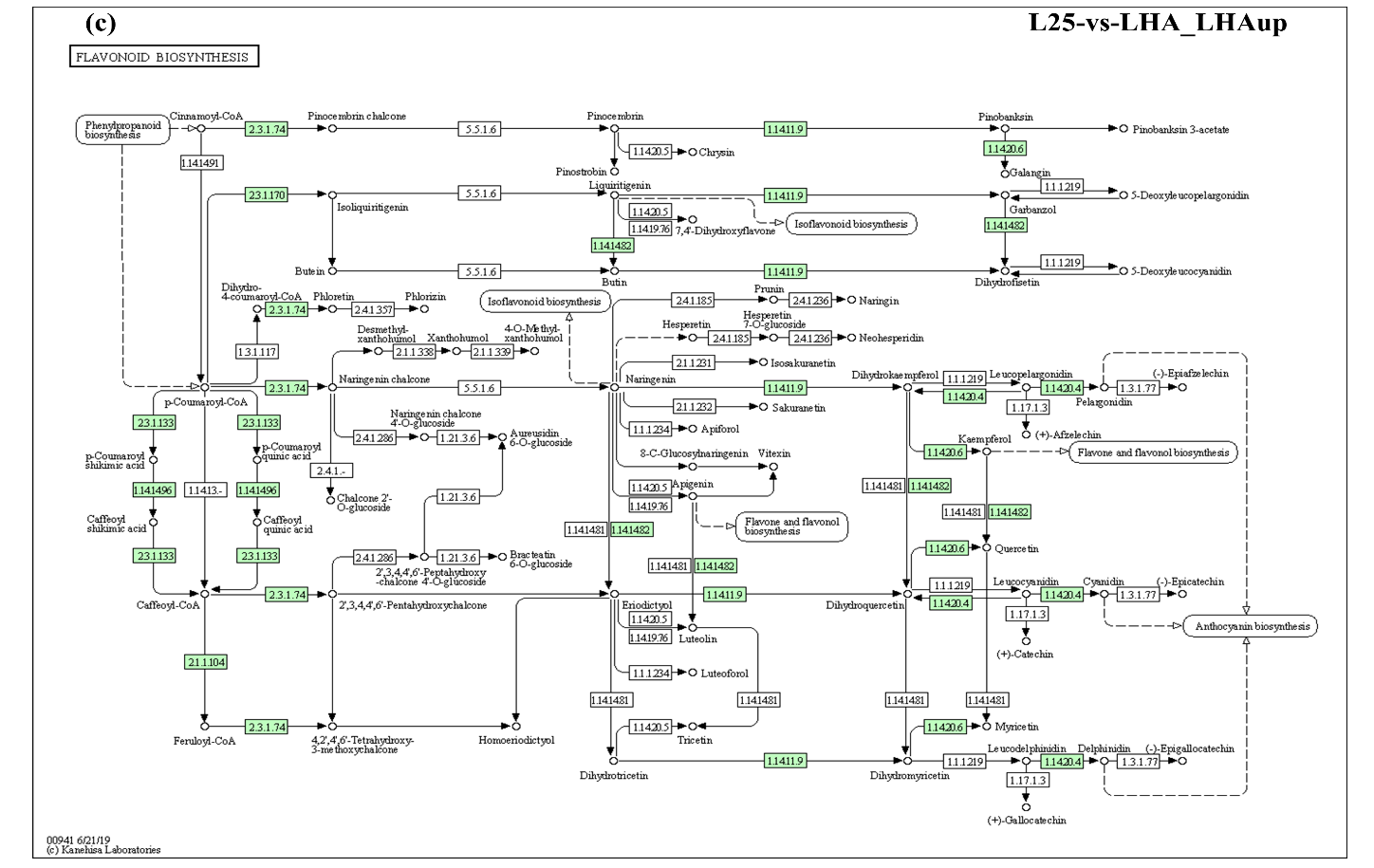


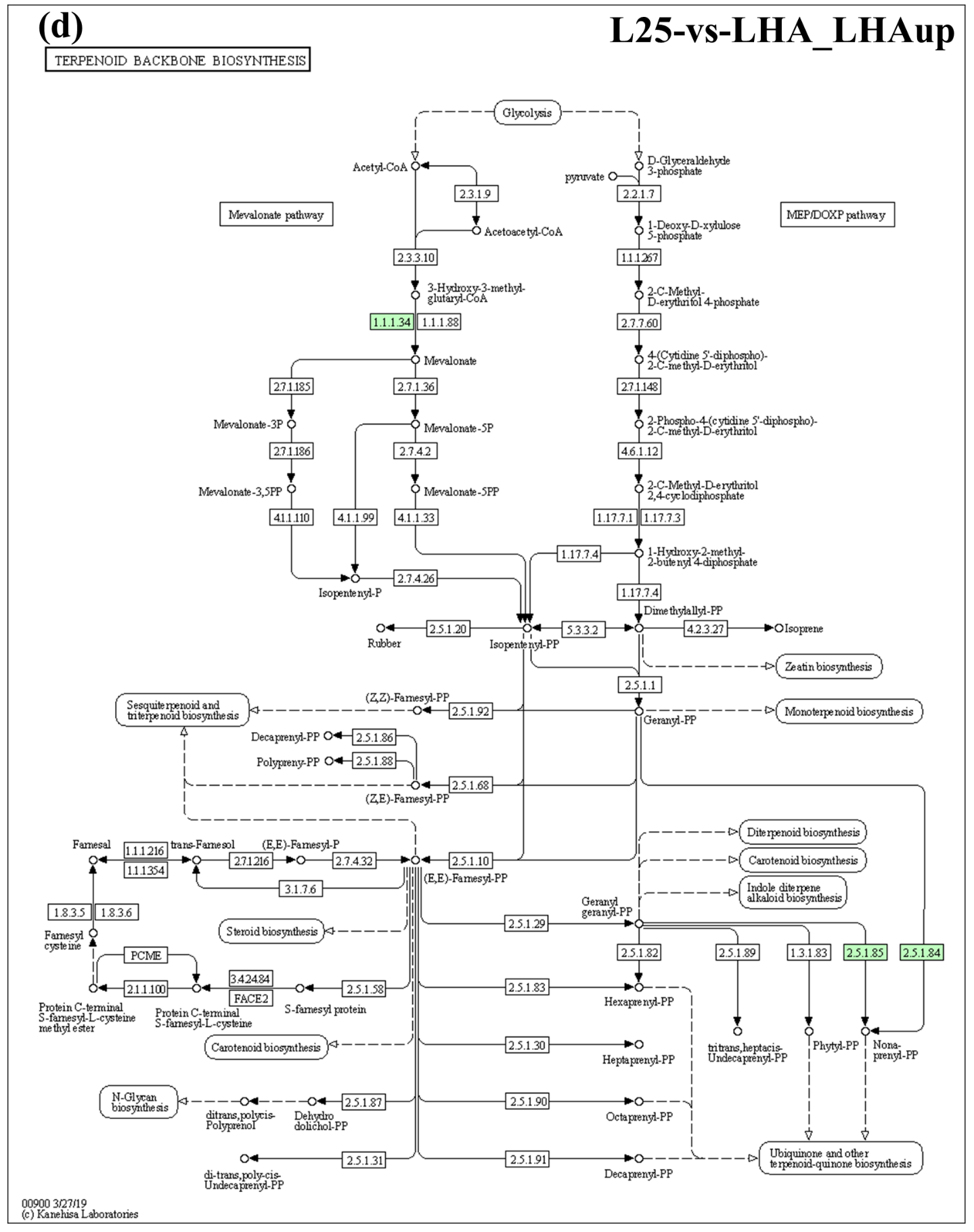


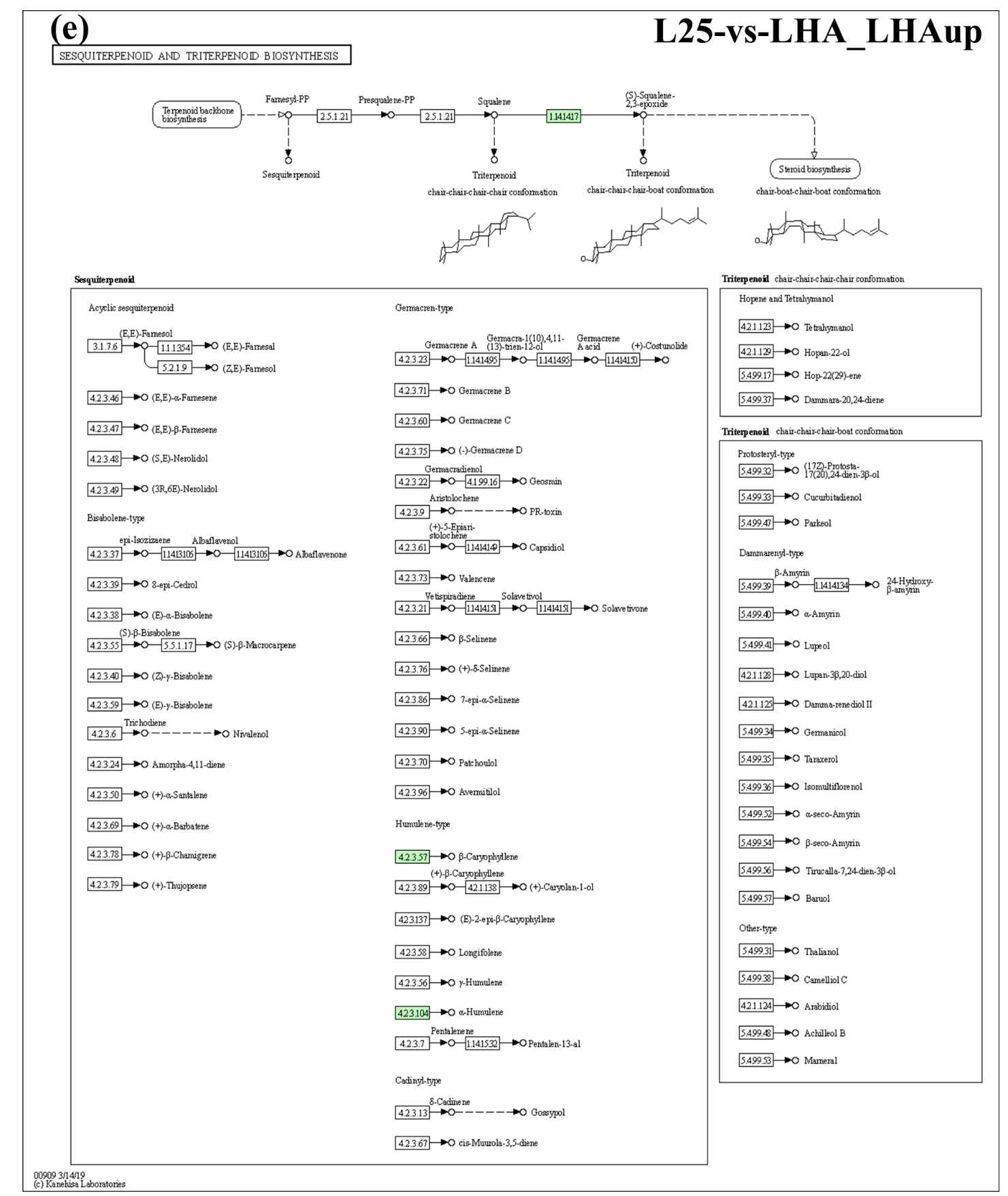


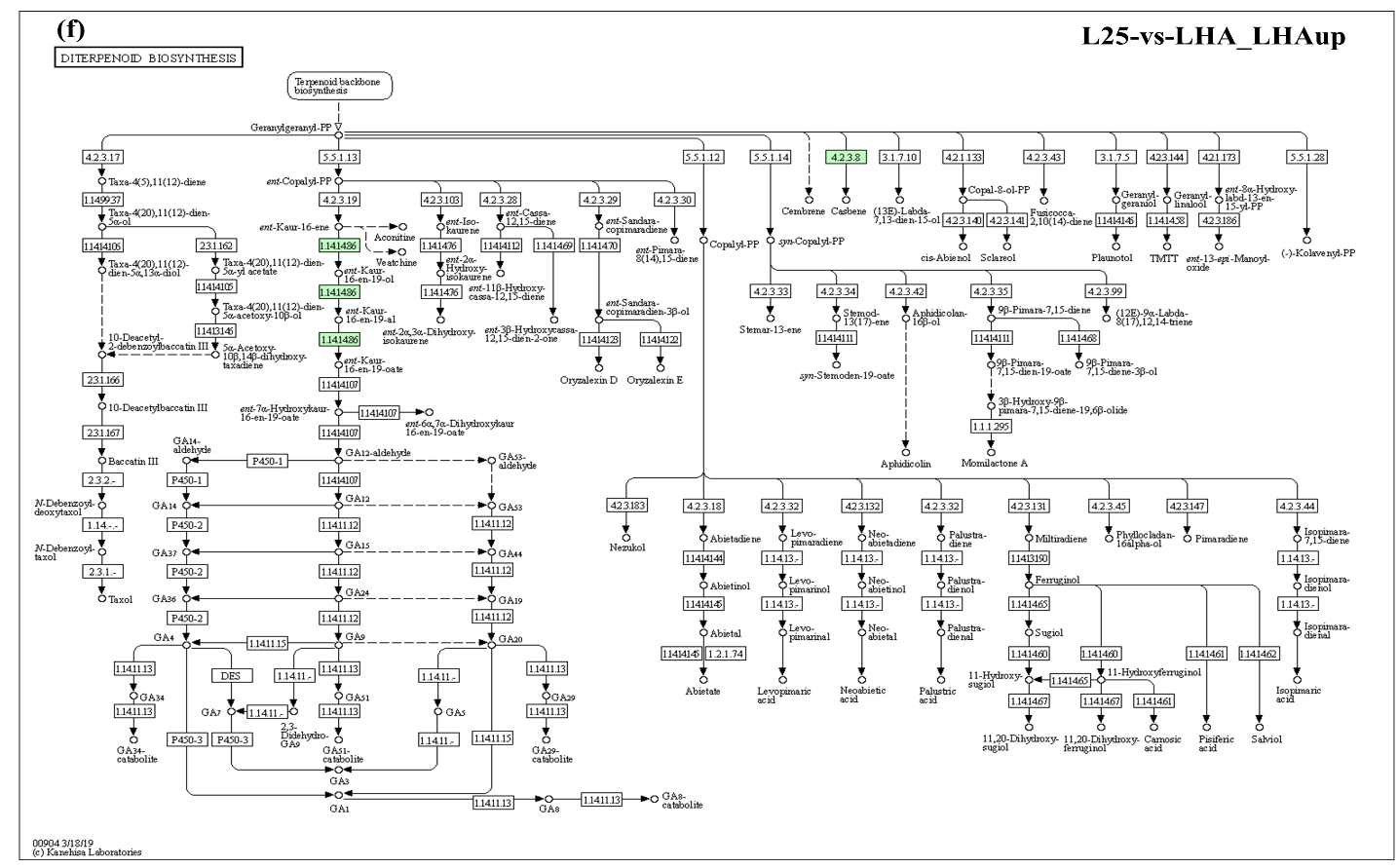


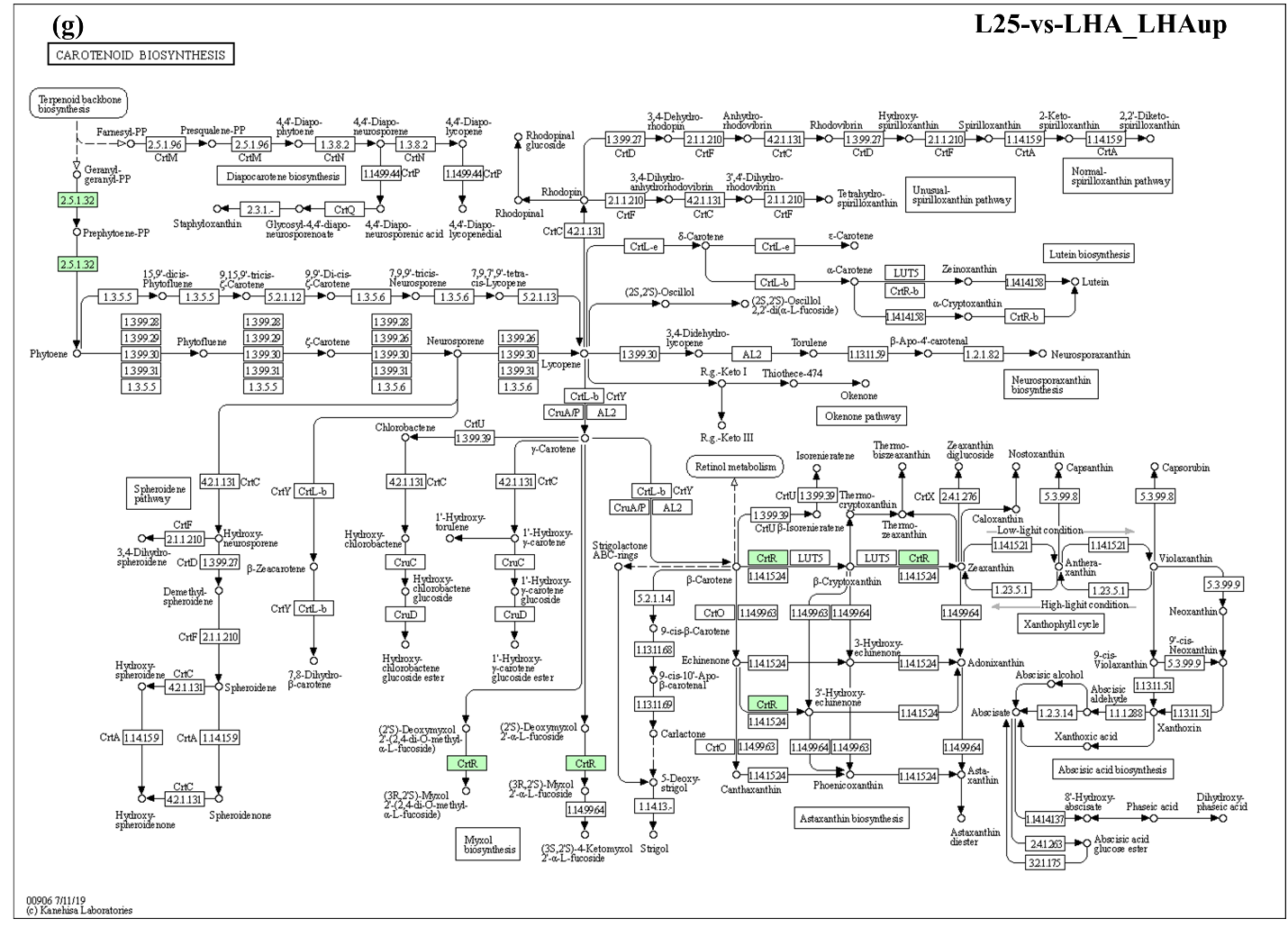


**Supplementary Figure S11. Pathway annotation of L25-vs-LHA_LHAup based on Kyoto Encyclopedia of Genes and Genomes (KEGG). (a-c)** Mapping of transcripts involved in lignin biosynthetic pathway and **(d-g)** transcripts involved in terpenoid pathway based on KEGG. Transcripts present in L25-vs-LHA_LHAup are annotated by "KEGG Mapper” (https://www.kegg.jp/kegg/tool/map_pathway.html)^4,5^ tool and marked with light green color boxes. White boxes indicate the transcripts that could not be identified in the pathway.


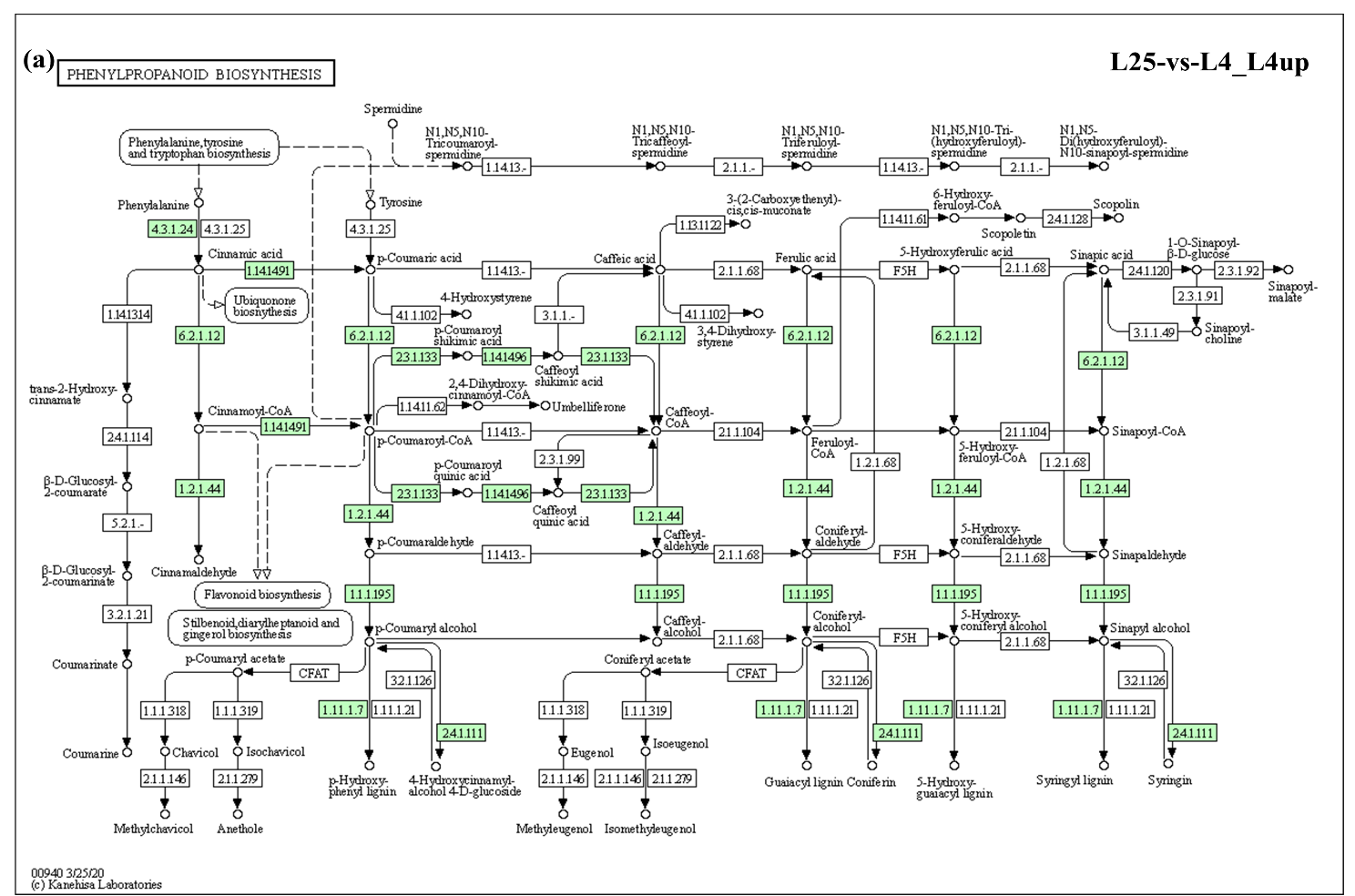


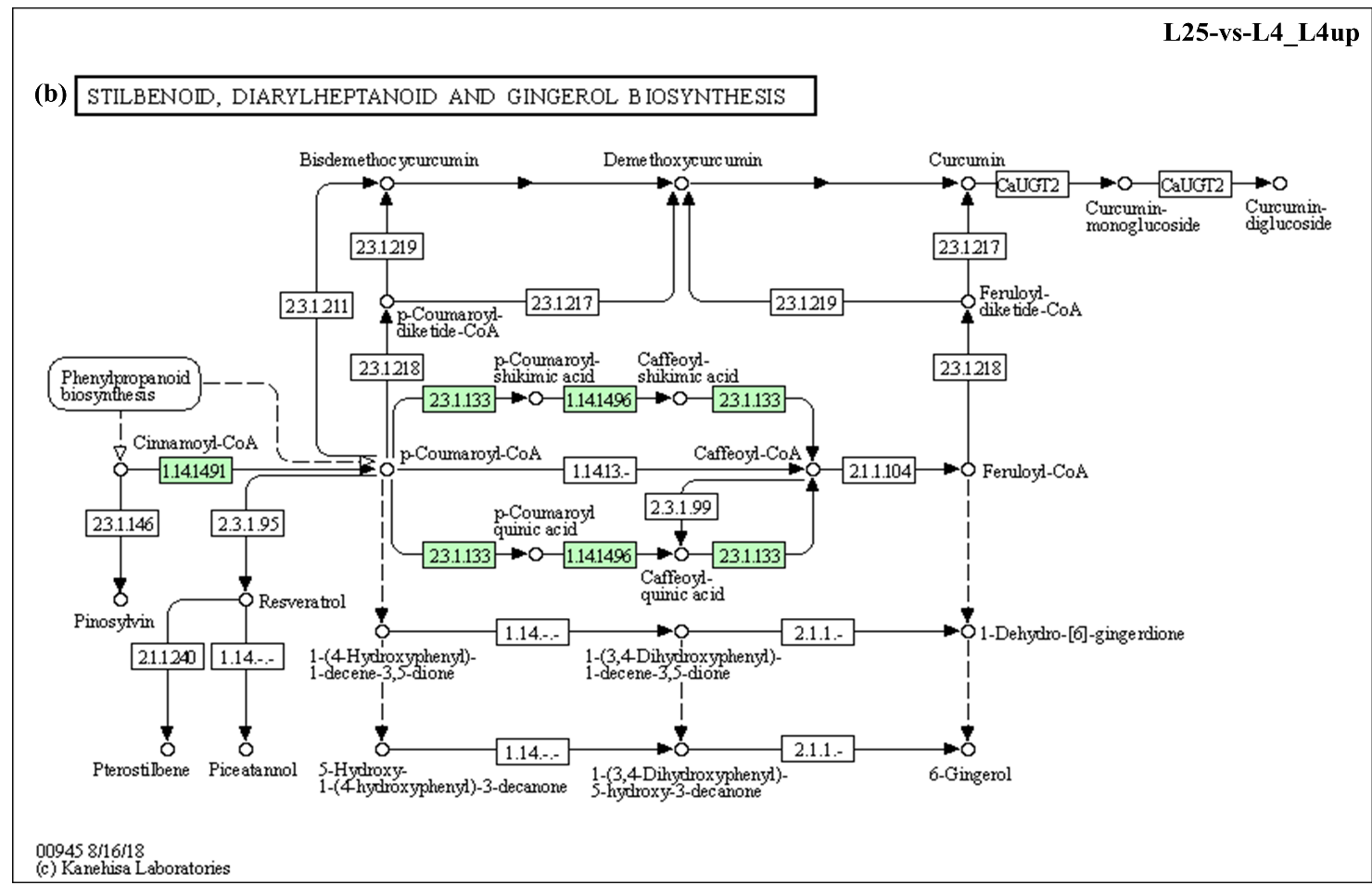


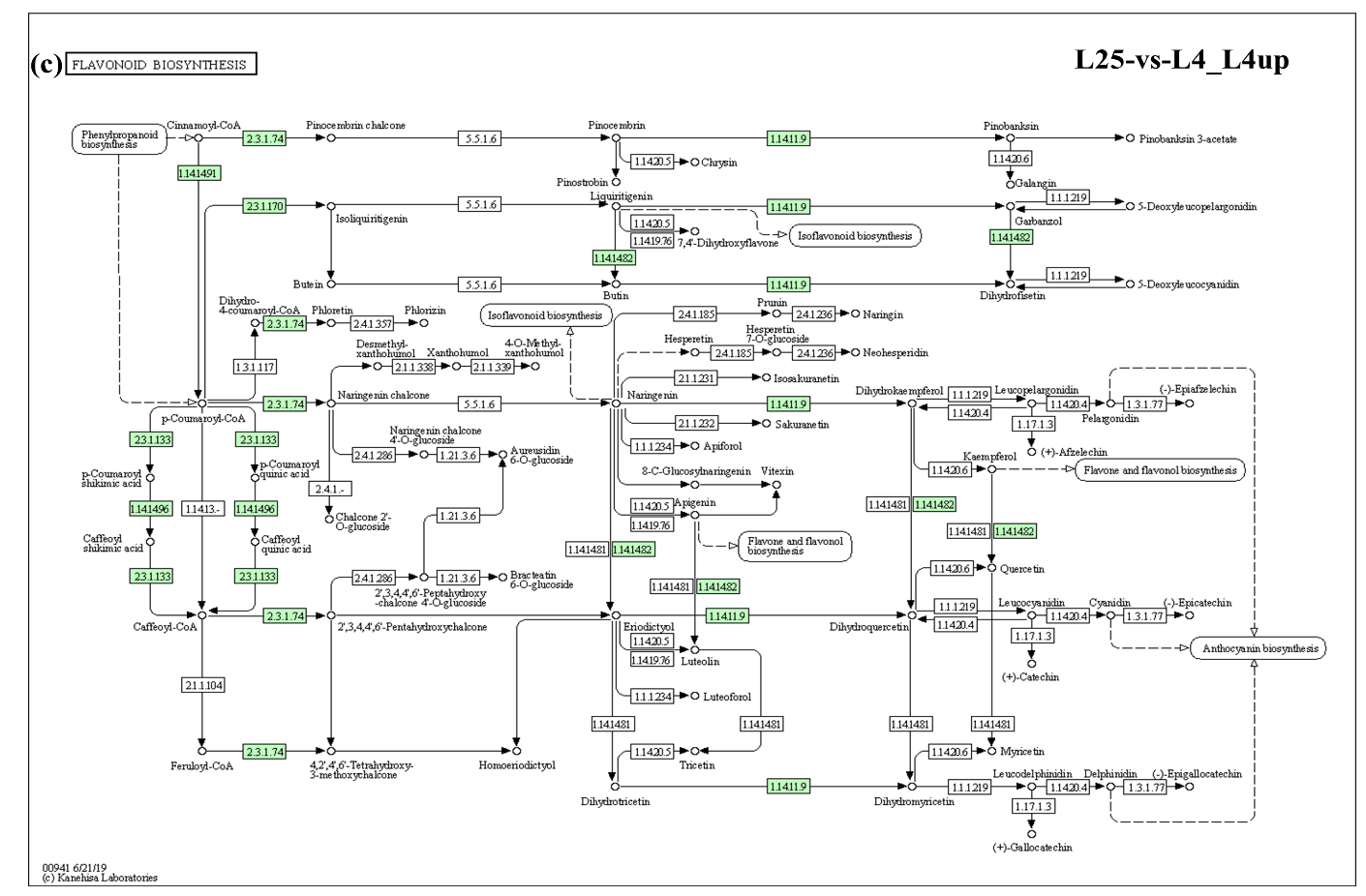


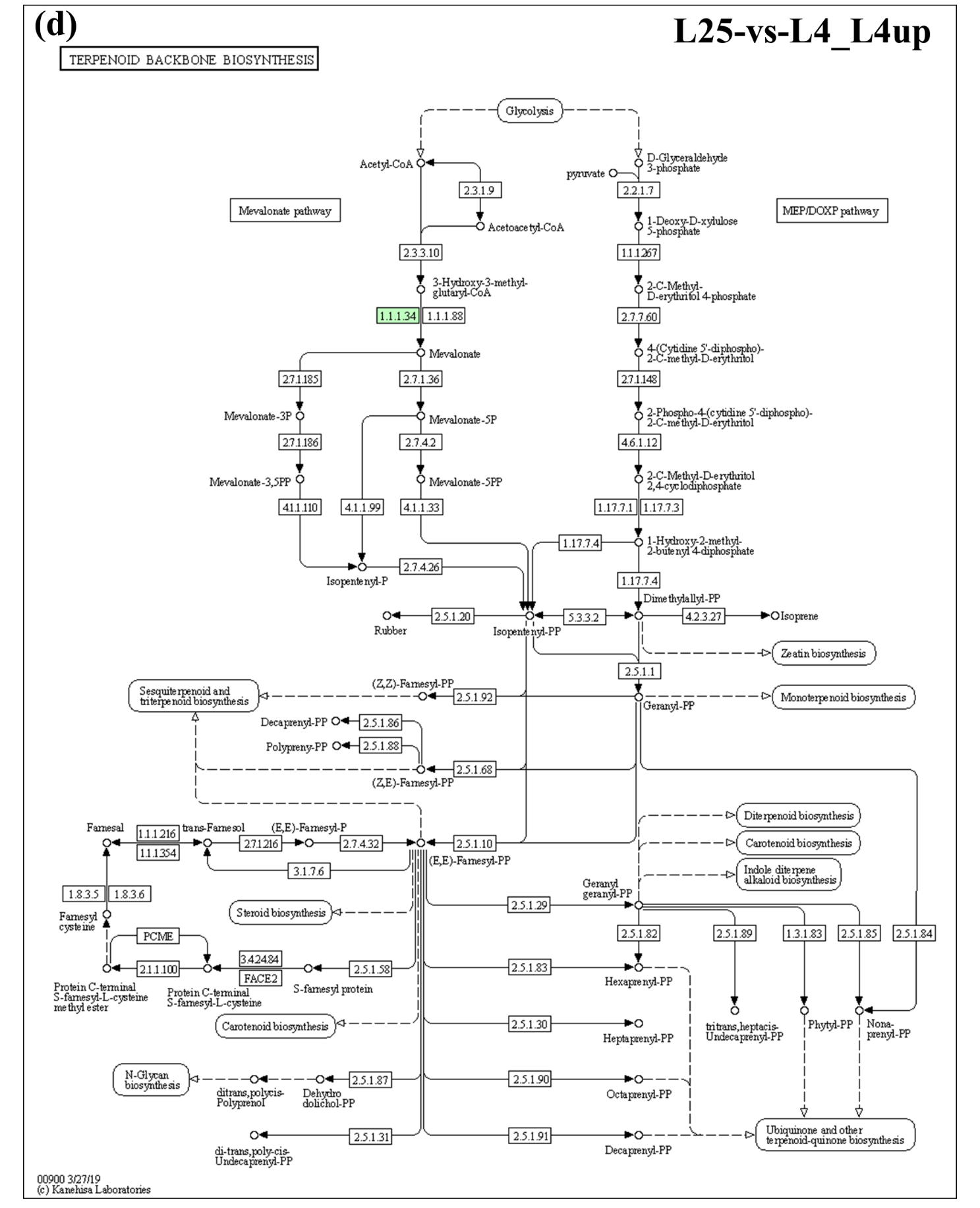


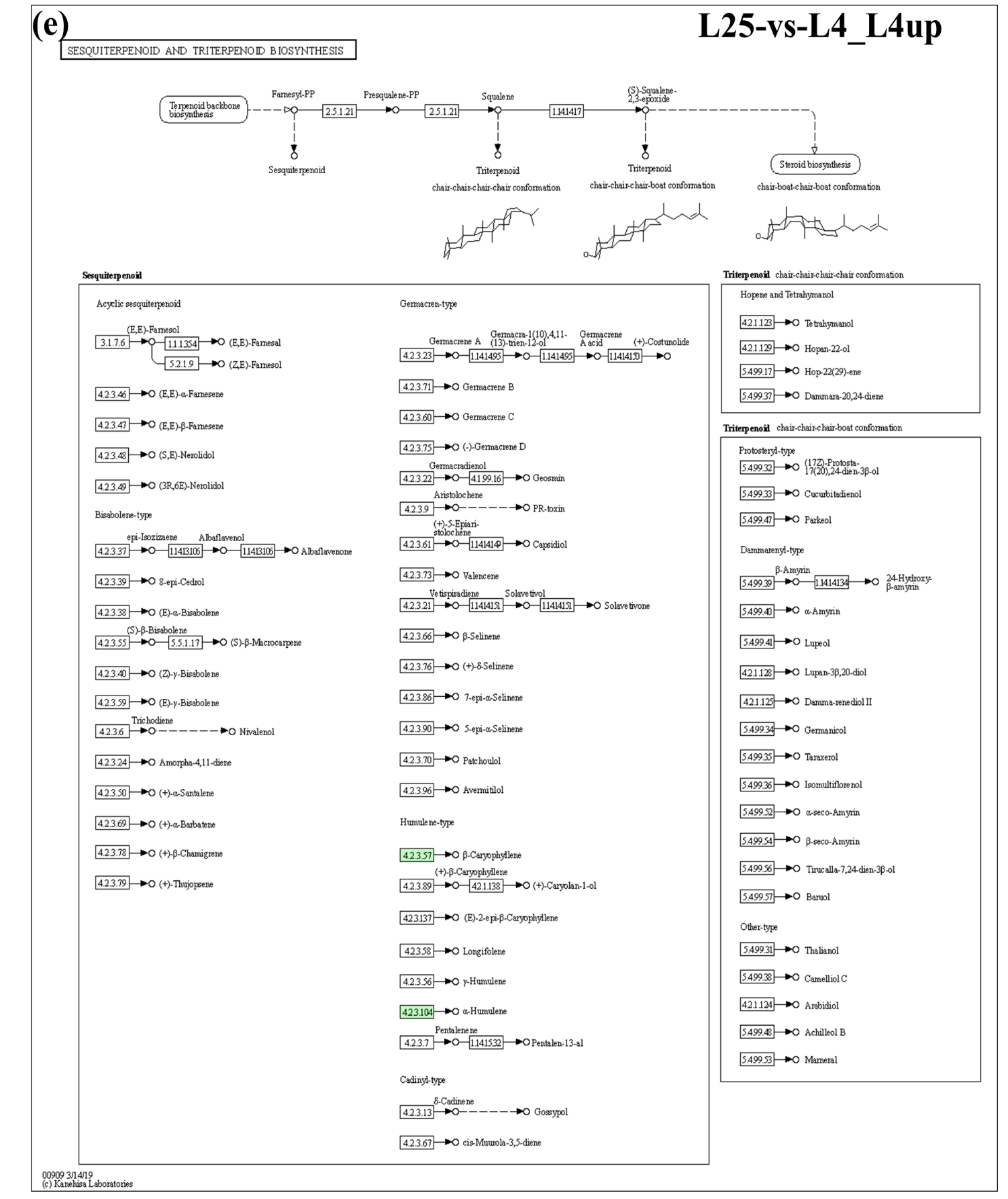


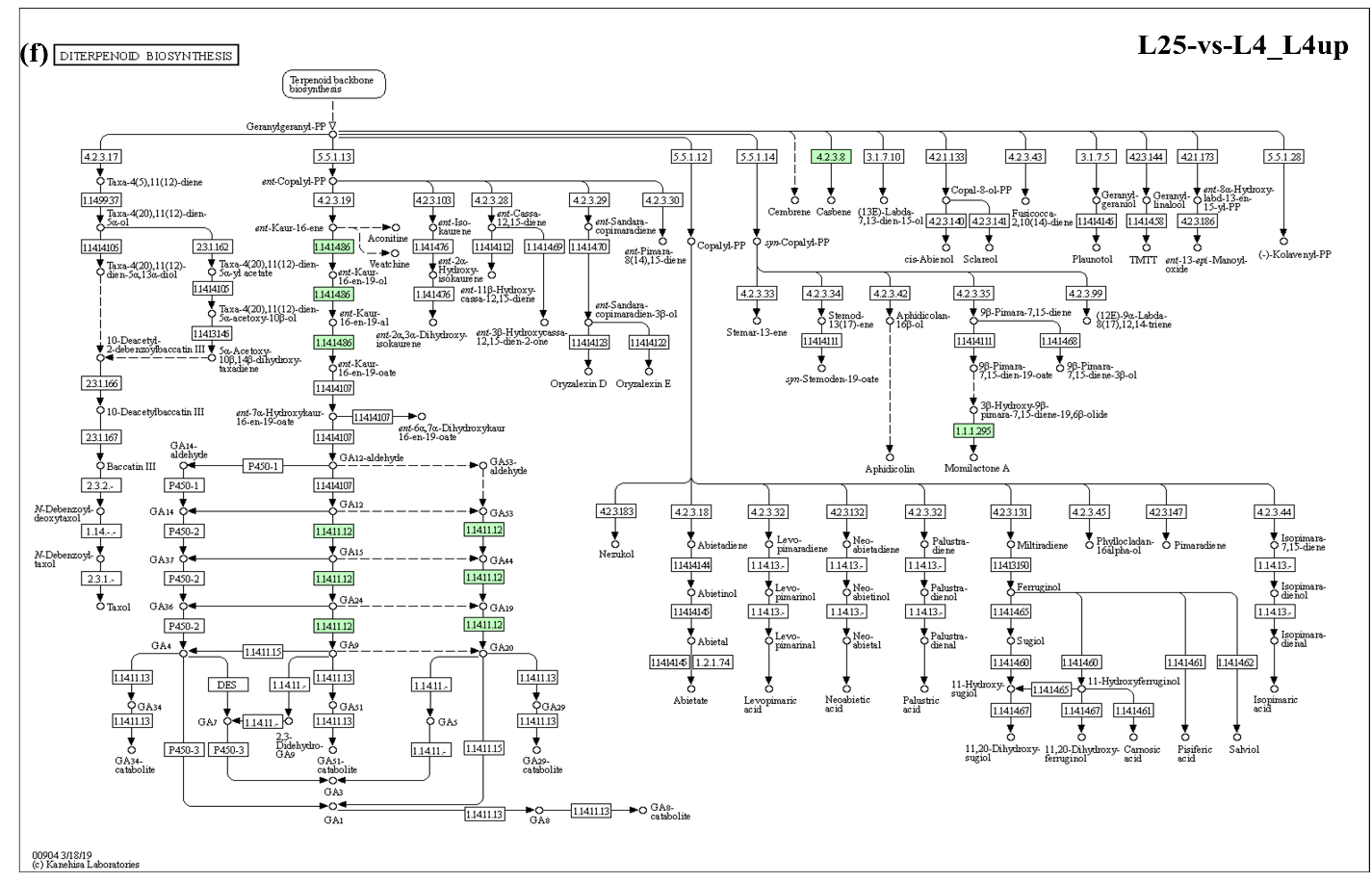


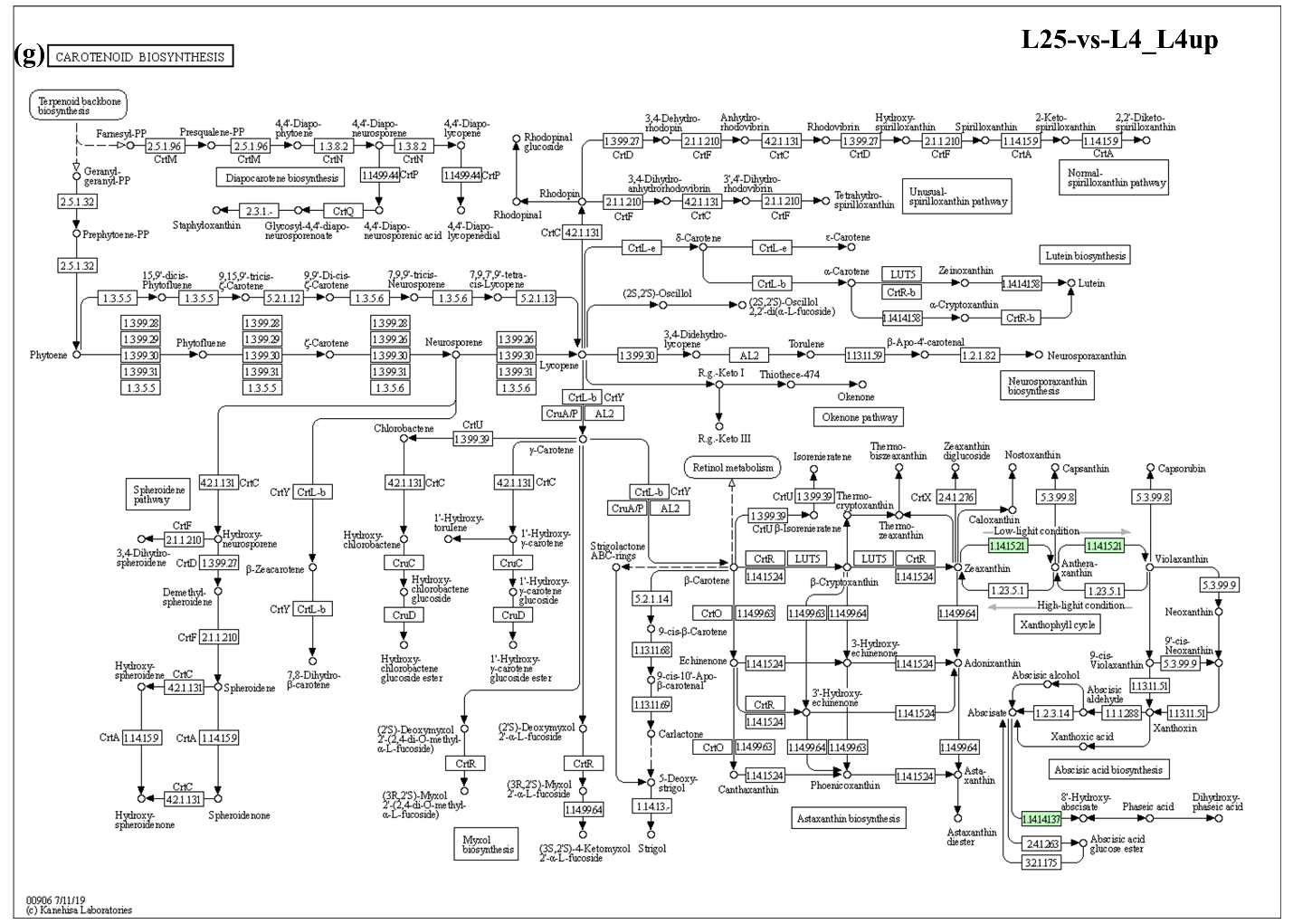


**Supplementary Figure S12. Pathway annotation of L25-vs-L4_L4up based on Kyoto Encyclopedia of Genes and Genomes (KEGG). (a-c)** Mapping of transcripts involved in lignin biosynthetic pathway and **(d-g)** transcripts involved in terpenoid pathway based on KEGG. Transcripts present in L25-vs-L4_L4up are annotated by "KEGG Mapper” (https://www.kegg.jp/kegg/tool/map_pathway.html)^4,5^ tool and marked with light green color boxes. White boxes indicate the transcripts that could not be identified in the pathway.
